# Dynamic PML protein nucleolar associations with persistent DNA damage lesions in response to nucleolar stress and senescence-inducing stimuli

**DOI:** 10.18632/aging.102248

**Published:** 2019-09-07

**Authors:** Terezie Imrichova, Sona Hubackova, Alena Kucerova, Jan Kosla, Jiri Bartek, Zdenek Hodny, Pavla Vasicova

**Affiliations:** 1 Department of Genome Integrity, Institute of Molecular Genetics of the Czech Academy of Sciences, Prague, Czech Republic; 2 Genome Integrity Unit, Danish Cancer Society Research Center, Copenhagen, Denmark; 3 Division of Genome Biology, Department of Medical Biochemistry and Biophysics, Karolinska Institute, Stockholm, Sweden; 4 Present address: Institute of Biotechnology, Czech Academy of Sciences, Prague-West, Czech Republic

**Keywords:** rDNA loci, super-resolution microscopy, time-lapse imaging, nucleolar segregation, DNA damage

## Abstract

Diverse stress insults trigger interactions of PML with nucleolus, however, the function of these PML nucleolar associations (PNAs) remains unclear. Here we show that during induction of DNA damage-induced senescence in human non-cancerous cells, PML accumulates at the nucleolar periphery simultaneously with inactivation of RNA polymerase I (RNAP I) and nucleolar segregation. Using time-lapse and high-resolution microscopy, we followed the genesis, structural transitions and destiny of PNAs to show that: 1) the dynamic structural changes of the PML-nucleolar interaction are tightly associated with inactivation and reactivation of RNAP I-mediated transcription, respectively; 2) the PML-nucleolar compartment develops sequentially under stress and, upon stress termination, it culminates in either of two fates: disappearance or persistence; 3) all PNAs stages can associate with DNA damage markers; 4) the persistent, commonly long-lasting PML multi-protein nucleolar structures (PML-NDS) associate with markers of DNA damage, indicating a role of PNAs in persistent DNA damage response characteristic for senescent cells. Given the emerging evidence implicating PML in homologous recombination-directed DNA repair, we propose that PNAs contribute to sequestration and faithful repair of the highly unstable ribosomal DNA repeats, a fundamental process to maintain a precise balance between DNA repair mechanisms, with implications for genomic integrity and aging.

## INTRODUCTION

One of the biological processes contributing to aging and age-related diseases is cellular senescence – a cell response to various stresses, characterized by protracted halt of cell cycle due to supra-threshold elevation of inhibitors of cyclin-dependent kinases (iCdk). Cellular senescence participates in aging by two main mechanisms: cell cycle arrest of progenitor cells, preventing tissue renewal; and secretion of pro-inflammatory molecules, leading to chronic inflammation and tissue deterioration (reviewed in refs. [1, 2]). Accordingly, elimination of senescent cells in mice has a positive effect on their health and lifespan [3, 4]. The upstream insults leading to senescence *via* iCdk of Kip and INK4 families are diverse, encompassing oncogene activation, oxidative or genotoxic stress, often involving cytokine signaling, phenomena commonly leading to DNA damage and persistent DNA damage response (DDR; reviewed in ref. [5]). The persistent DDR due to irreparable or perpetual DNA damage is thought to be the main mechanism behind most forms of cellular senescence. The nature of this senescence-associated DNA damage seems to be complex and multifactorial though irreparability of telomeres is the factor most frequently cited [6, 7]. A decade ago, it has been proposed that rDNA instability is the major determinant of life-span in budding yeast [8, 9]. Recently, the direct evidence that damage of ribosomal DNA (rDNA) loci can also cause senescence has been reported [10, 11].

Nucleolus is a membrane-less organelle formed around the active rDNA repeats through a biophysical phenomenon known as liquid-liquid phase separation [12]. The main function of this compartment is ribosome biogenesis; however, in recent years, the role of nucleolus in cellular stress responses has been increasingly recognized. In short, various stress stimuli deregulate ribosome biogenesis, which results in activation of multiple nucleolus-associated molecular pathways that cause p53-dependent and -independent cell cycle arrest (reviewed in refs. [13–16]). Dependent on cellular context, this cell-cycle arrest may ultimately lead to, or reinforce, senescence [17, 18].

The PML is a structural component of specific nuclear compartment termed PML nuclear bodies (PML NBs; [19]) that is comprised of hundreds of proteins and involved in multitude of cellular functions such as transcription, posttranslational modifications, protein sequestration and degradation, antiviral response, DNA repair, cellular senescence and apoptosis (reviewed in ref. [20]). PML NBs co-associate with late (irreparable) DNA damage foci [21–24] characteristic for senescent cells [25–27]. The exact function of PML and PML NBs in DNA repair is still under investigation, however, emerging evidence indicates their involvement in DNA repair by homologous recombination [25, 28].

Replicative senescence of human mesenchymal stem cells is associated with interaction of PML with the surface of the nucleolus [29]. The association of PML with nucleolus was also observed after treatment of various cell types with several senescence-inducing stimuli, for instance mouse and human embryonic fibroblasts with doxorubicin and γ-irradiation (IR) and human mesenchymal stem cells (hMSC) with actinomycin D (AMD; [29–32]). Strikingly, the association of PML with nucleoli of most cancer cell lines is rather low [29]. Two general structural types of PML association with the nucleolus were described after AMD treatment of hMSC [29]. The first type is characterized by association of PML with the border of a segregated nucleolus during functional inactivation of DNA-dependent RNA polymerase I (RNAP I). The second type termed PML nucleolus-derived structure (PML-NDS; [29]) is localized tightly to reactivated/active nucleolus as a structure of sub-nucleolar size accumulating some nucleolar proteins and appearing in increasing frequency with time after AMD removal during pre-rRNA transcription recovery. Based on indirect evidence, it was proposed that these two structures resemble two developmental stages of the same process triggered by nucleolar stress, however, no direct proof for such concept has been provided.

In this study we employed a long-term live-cell time-lapse imaging with 3-D image reconstruction and super-resolution microscopy to provide evidence that the PML nucleolar compartment is dynamic, including several structural transition states that are associated with inactivation and reactivation of nucleolar transcription during nucleolar stress, respectively. All forms of PML nucleolar associations including the late forms of PNAs, the PML-NDS, colocalized with DNA damage markers indicating a role of PML in DNA repair of nucleoli-associated rDNA loci. PML-NDS may last for very long periods of time despite some of them can eventually lose the DNA damage signal. The persistence of PML-NDS associated with DNA damage suggests that irreparable damage of rDNA loci might contribute to the pool of damaged DNA responsible for maintenance of the cellular senescent phenotype.

## RESULTS

### Senescence-inducing doses of doxorubicin provoke structurally variable association of PML with nucleoli

**Figure 1:**
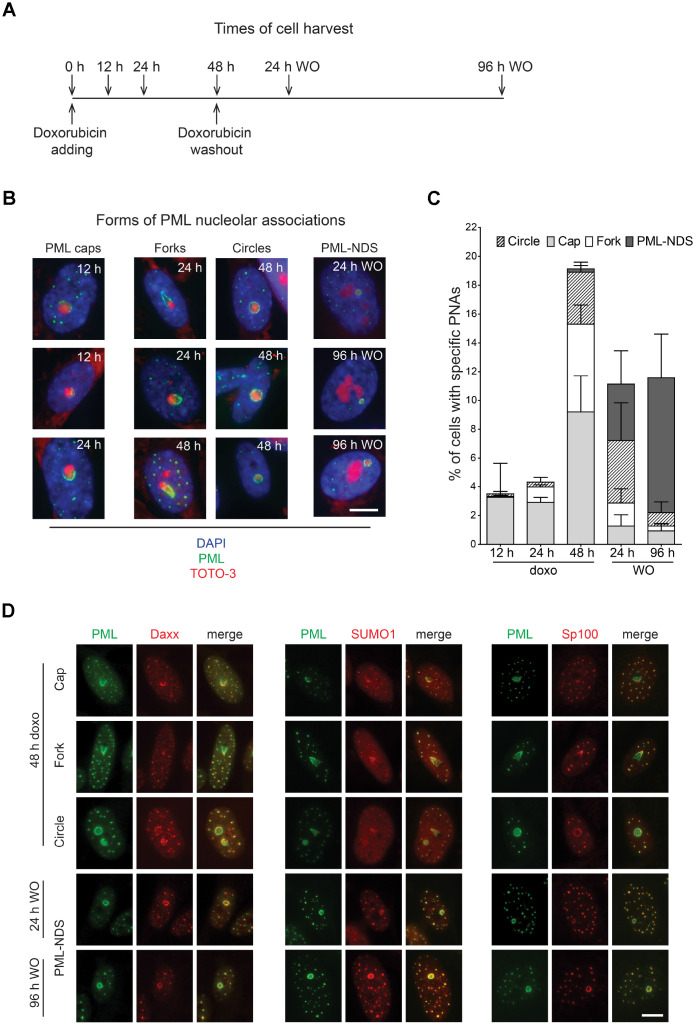
**Time-dependent differences in structural forms of PML nucleolar associations induced by doxorubicin.** As schematically depicted (**A**), RPE-1^*hTERT*^ were treated with 0.75 μM doxorubicin and diverse types of PNAs were quantified by analysis of microscopic images in several time-points as indicated in the scheme. (**B**) Representative images of structural categories of PNAs obtained by wide-field indirect immunofluorescence microscopy of nuclei immunostained for PML (green). Nuclear and nucleolar compartments were visualized with DAPI (blue) and TOTO-3 (red), respectively. The images were captured with 63×/1.4 objective. Bar, 10 μm. (**C**) The percentage of cells containing specific structural subtypes of PNAs categorized as 'circles', 'caps', 'forks' and 'PML nucleoli-derived structures' (PML-NDS) was estimated. Over 200 cells in three biological replicates were evaluated for each time-point. Results are presented as a mean ± s.d. (**D**) Indirect immunofluorescence showing the colocalization of PNAs with proteins of PML nuclear bodies (PML-NBs). PML (green) and PML-NBs proteins (red) are visualized with respective antibodies, the nucleus was stained with DAPI (blue) and the nucleolus with TOTO-3 (cyan). The images were captured with 63×/1.4 objective. Bar, 10 μm.

Several classes of compounds including topoisomerase and RNAP I inhibitors can induce the association of PML with nucleolus in human non-cancerous cells ([29–32] and our unpublished data). For the purpose of this study, we selected the topoisomerase II inhibitor doxorubicin used clinically as a chemotherapeutic agent for treatment of several human malignancies as a DNA damaging agent, and the human telomerase-immortalized retinal pigment epithelial cell line RPE-1^*hTERT*^ as an easily manipulatable non-transformed model. At first, we determined the senescence-inducing dose of doxorubicin (0.75 μM) in RPE-1^*hTERT*^ as the treatment with the highest ratio of the extent and variability of PNAs to cell death (Supplementary Figure 1A). After two weeks, RPE-1^*hTERT*^ cells treated with this dose of doxorubicin developed cellular senescence as shown by loss of proliferation, presence of DNA damage detected as γH2A.X foci, characteristic morphological changes, and positivity for senescence-associated β-galactosidase staining (Supplementary Figure 1B–1E). Senescent cells survived at least for four weeks without regaining cell proliferation (data not shown). The formation of diverse PML nucleolar associations was followed by wide field microscopy of formaldehyde-fixed cells harvested at several time points after doxorubicin addition (12, 24 and 48 hours) and after doxorubicin washout (24 and 96 hours; see scheme in Figure 1A). The relative presence of specific forms of PNAs resembling caps, forks, circles and PML-NDS (termed according to their 2-D appearance) clearly changed during the time of doxorubicin treatment and washout (Figure 1B, 1C). The proportion of PML ‘cap-like’ nucleolar structures dominated in the 12-hour sample and coincided with the stress-induced elevation of the PML protein levels (note, unperturbed RPE-1^*hTERT*^ cells showed a very low level of PML and number of PML NBs). The appearance of PML ‘fork-like’ and ‘circle-like’ nucleolar structures followed the PML caps as could be detected in the 48-hour samples and these structures persisted even after doxorubicin removal. PML-NDS were almost undetected until 48 hours after doxorubicin addition, however, their presence strongly increased after doxorubicin washout and was associated with the decrease of other PNAs forms as can be observed at 96 hours after doxorubicin removal. All forms of PNAs were positive for other constituents of PML NBs such as Sp100, Daxx and SUMO1 (Figure 1D). Note, not all cell nucleoli contained PNAs at the same time and some cells contained nucleoli with different forms of PNAs simultaneously, indicating asynchronous changes in functional states of individual nucleoli (Supplementary Figure 2A, 2B). The formation of PNAs was not restricted to doxorubicin-treated RPE-1^*hTERT*^ cells, as human mesenchymal stem cells (hMSC) or normal diploid BJ fibroblasts showed similar extents and patterns of PNAs formation (Supplementary Figure 2C).

Altogether, this data shows that PML protein can associate with nucleoli during senescence-inducing stress to form structurally variable accumulations at nucleolar periphery. This newly formed PML nucleolar compartment was relatively stable, however, not all nucleoli of a given cell were affected at the same time indicating their asynchronous response to the stress.

### Structural transitions of PML nucleolar associations follow the activity state of the nucleolus

**Figure 2:**
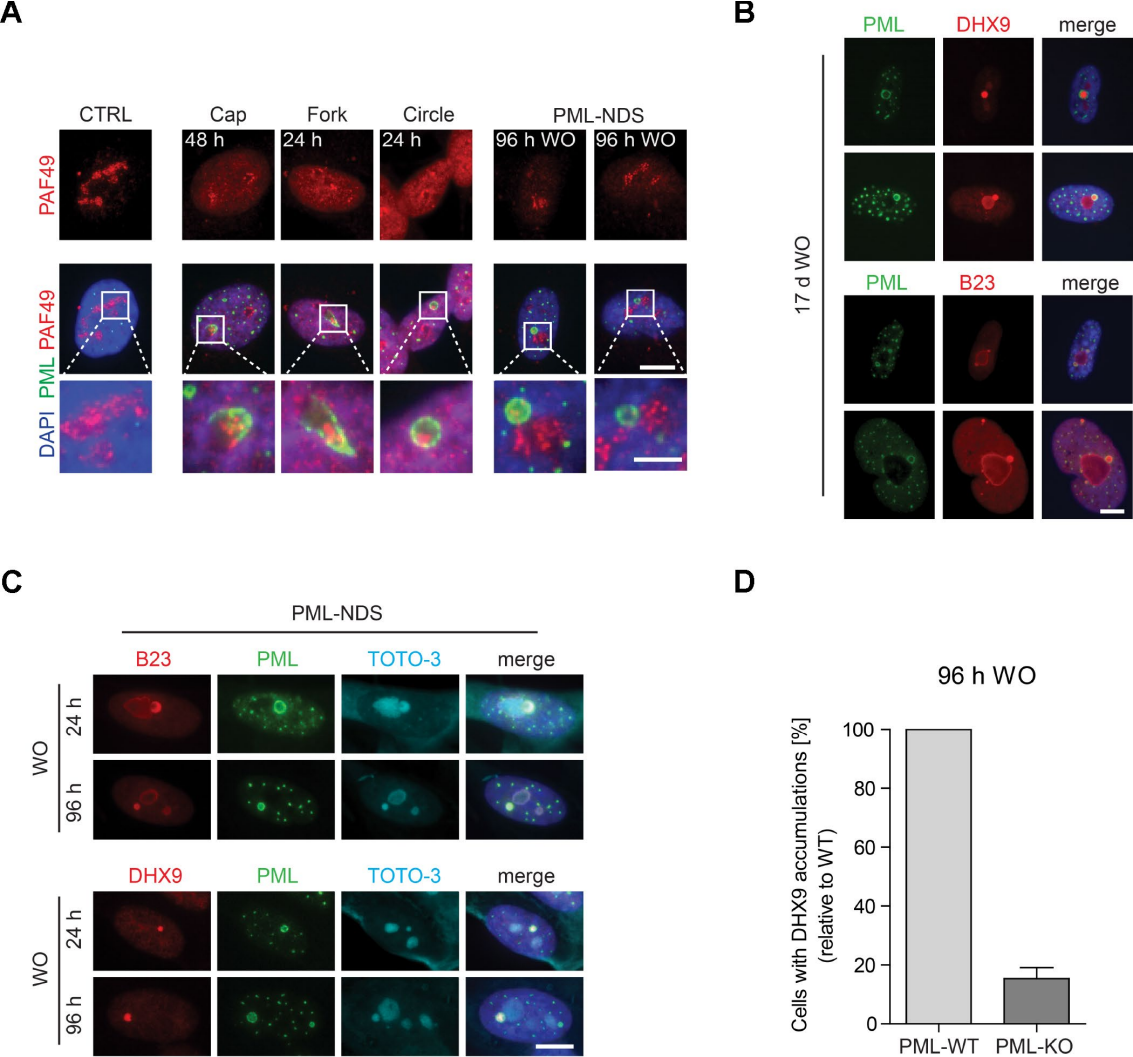
**The association of specific subtypes of PNAs with different functional states of nucleoli.** (**A**) The activity of RNAP I, evaluated as relocalization (segregation) of RNAP I subunit PAF49 and the presence of specific forms of PNAs were visualized by wide-field immunofluorescence microscopy of PAF49 (red) and PML (green) in RPE-1^*hTERT*^ during different time-points of doxorubicin-treatment (0.75 μM) and its removal (WO). The insets show selected nucleoli with different states of RNAP I. Bars, 10 μm for whole cells and 4 μm for insets. (**B**) Long-term persistence (17 days after doxorubicin removal) of PML-NDS marked by PML (green) with accumulations of nucleolar proteins DHX9 and B23 (both in red). Bar, 10 μm. (**C**) Accumulation of B23 and DHX9 inside PML-NDS was visualized by immunostaining with respective antibodies (accumulated proteins – red, PML – green). Nuclei (**A**–**C**) and nucleoli (**C**) were visualized by DAPI (blue) and TOTO-3 (cyan), respectively. The images were captured with 63×/1.4 objective. Bar, 10 μm. (**D**) RPE-1^*hTERT*^ PML-WT and PML-KO cells were treated with 0.75 μM doxorubicin for 48 hours; after that doxorubicin was removed and the cells were further cultured. 96 hours after doxorubicin washout the cells were fixed, stained with antibodies against DHX9 and PML and imaged with the Olympus ScanR microscope. The occurrence of cells with DHX9 accumulations were analyzed by the ScanR analysis software. The relative occurrence of cells with accumulations of DHX9 is shown. Two biological replicates were evaluated. Results are presented as a mean ± s.d.

Doxorubicin inhibits rDNA transcription resulting in nucleolar segregation [33], a well-established nucleolar restructuring process ensuing inhibition of RNAP I activity [34]. To link specific types of PNAs to the RNAP I activity, the distribution of RNAP I subunit PAF49 was followed after the exposure of cells to doxorubicin by indirect immunofluorescence. As shown in Figure 2A, the presence of PML caps, forks and circles was always linked to the PAF49 segregated to nucleolar periphery pointing to their association with the RNAP I inhibition. In fact, the PML cap-like structures tightly co-associated with the PAF49 segregated into a structure termed nucleolar cap [34]. This association was even more apparent in the PML fork-like PNAs where PAF49 localized directly in between the fork arms (Figure 2A).

The PML-NDS were usually present next to the active nucleolus, indicating their association with a recovery of pre-rRNA transcription. Indeed, the gradual onset of RNAP I activity after doxorubicin washout was verified by 5-fluorouridine (5-FUrd) incorporation (see Supplementary Figure 3A, 3B) when a fraction of 5-FUrd-positive nucleoli was detected already 3 hours after doxorubicin washout. 24 hours after drug removal, most of the nucleoli were 5-FUrd-positive indicating almost complete restoration of rDNA transcription. Notably, PML-NDS accumulating B23 and DHX9 were present in the cells even 17 days after doxorubicin washout (Figure 2B) indicating their strong association with long-term cell-cycle arrest and senescent state [29].

The characteristic feature of the PML-NDS, distinguishing them from the large PML NBs, is an accumulation of nucleophosmin (B23), and DNA helicase II (DHX9) (Figure 2B, 2C; [29]). Sequestration of these proteins into PML-NDS is most likely a selective process, as several other nucleolar proteins, such as fibrillarin, Nopp140, PAF49 and UBF, localized into PML-NDS only partly or not at all (Supplementary Figure 4). To test whether the genesis of these accumulations requires PML, we utilized the RPE-1^*hTERT*^ cells in which *PML* had been completely ablated by gene knock-out (PML-KO; [25]). The PML wild-type (PML-WT) and PML-KO cells were stained for DHX9 and subjected to quantitative analysis by Olympus ScanR at 96 hours after doxorubicin washout, i.e. at a time-point when PML-NDS were readily detectable in PML-WT cells. As shown in Figure 2D, a five times higher number of PML-WT cells with DHX9 spots – compared to PML-KO cells – was detected, indicating a functional role of PML in the formation of PML-NDS. It should be emphasized that the DHX9 accumulations identified in PML-KO cells were overall less intense and of irregular shape (Supplementary Figure 5A), suggesting they reflected rather random spots of higher DHX9 signal accumulation, unlikely representing the proper PML-derived DHX9 accumulations. To elucidate whether DHX9 might be primed for degradation in PML-NDS, we measured the total protein level of DHX9 in PML-WT and PML-KO cells at several time-points after doxorubicin addition and found that even though DHX9 was gradually degraded after doxorubicin treatment, the degradation was not dependent on PML, as there was no significant difference in DHX9 level between PML-WT and PML-KO cells (Supplementary Figure 5B).

Altogether, these findings indicate that the structural changes of PML nucleolar compartment reflect the state of rDNA transcription inhibited by the topoisomerase inhibitor doxorubicin, when PML can associate with either of the two distinct nucleolar states: inactive (segregated) or reactivated nucleolus. The latter is represented as small relatively long-lived nucleolar accessory structures (PML-NDS) with PML-dependent accumulation of specific proteins such as DHX9 and localization juxtaposed to the reactivated nucleolus.

### High-resolution 3-D structures of individual PNAs

**Figure 3:**
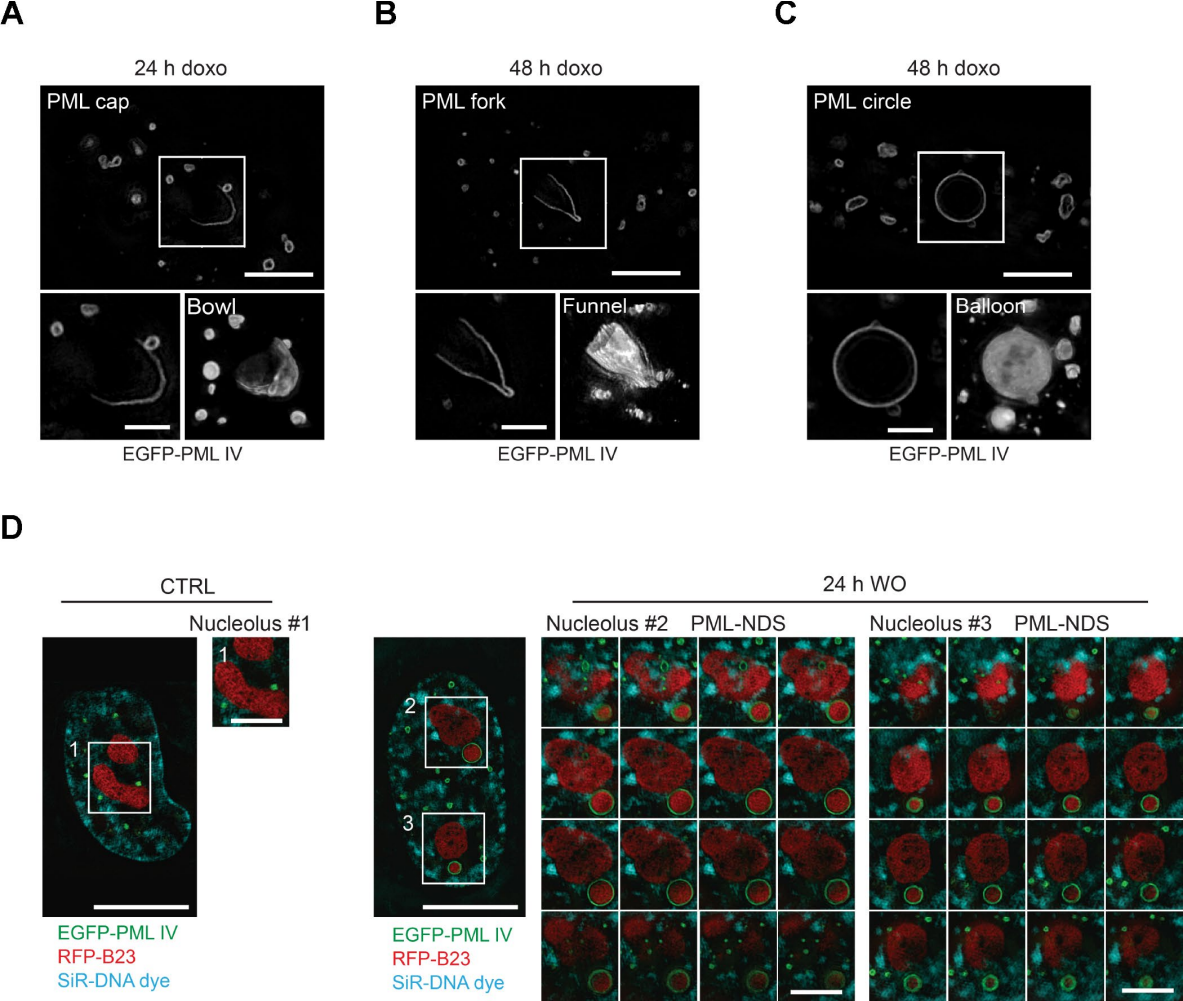
**Three-dimensional reconstruction of structural subtypes of PNAs.** High resolution live-cell structured illumination microscopy of the cap- (**A**), fork- (**B**) and circle-like (**C**) PNAs of RPE-1^*hTERT*^ cells stably expressing the EGFP-PML IV isoform harvested 24 and 48 hours after doxorubicin-treatment. Central layer of the whole cell (upper images; bar, 5 μm) and central layer of respective type of PNAs (lower-left images; bar, 2 μm) are shown together with reconstructed 3-D images (lower-right images) from 28 (cap), 30 (fork) and 54 (circle) layers using ImageJ 3D viewer plugin. (**D**) High resolution live-cell SIM images of RPE-1^*hTERT*^ stably expressing EGFP-PML IV and RFP-B23. A control untreated cell (left) and a cell containing PML-NDS, imaged 24 hours after doxorubicin washout (right). The central layer of whole cells (bar, 10 μm) are shown together with three insets of nucleoli (1 layer for the nucleolus of control cell and 16 layers for the nucleoli with adjacent PML-NDS; bar, 4 μm). DNA was labeled with SiR-DNA dye.

To obtain detailed information about a 3-D structure of PNAs, we performed live-cell imaging analysis of RPE-1^h*TERT*^ cells stably expressing EGFP-PML IV, a PML isoform with the highest capacity to form PNAs after doxorubicin [31], using structured illumination microscopy (SIM) after doxorubicin treatment. We noted that in 3 dimensions, the structures represented in 2-D as caps, circles and forks were in fact bowls and hollow balloons and funnels, respectively (Figure 3A–3C and Supplementary Videos 1–3). Importantly, we could clearly differentiate the two distinct shapes, balloons and funnels, confirming that the formerly observed circles and forks are not the same type of PNA seen from different angles but rather two structurally separate types of PNAs.

To unambiguously dissect the position of the PNAs relative to nucleoli, we employed SIM on doxorubicin-treated live cells expressing ectopic EGFP-PML IV, and RFP-B23 as a nucleolar marker. In this setting, we could observe that both the balloons and the funnels undeniably enclose the nucleolus (Supplementary Figure 6A, 6B). Furthermore, we confirmed that also PML-NDS contain nucleolar material. The RFP-B23 signal inside PML-NDS was very dense without observable small pores; whereas the RFP-B23 signal in adjacent nucleoli was less intense with numerous pores and holes, resembling the nucleoli of untreated cells (Figure 3D). On the other hand, the density and structure of RFP-B23 in the PML-NDS was comparable with RFP-B23 enclosed in funnel- and balloon-like PNAs indicating their similar metabolic state (Supplementary Figure 6A, 6B). To exclude artifacts that could result from the use of ectopically expressed fluorescently tagged PML protein, we employed stimulated emission depletion (STED) super-resolution microscopy of fixed cells with endogenous PML and B23 stained by indirect immunofluorescence. Although the sample preparation caused reduction of cell volume and compromised the continuity of the PML shell, we could still detect the PML signal around the whole nucleolus, confirming our SIM data (Supplementary Figure 6C, 6D).

Overall, using two super-resolution techniques, we confirmed that the PML can form several structurally variable interactions with the nucleolar periphery.

### Evolution and fate of ‘early’ PNAs

**Figure 4:**
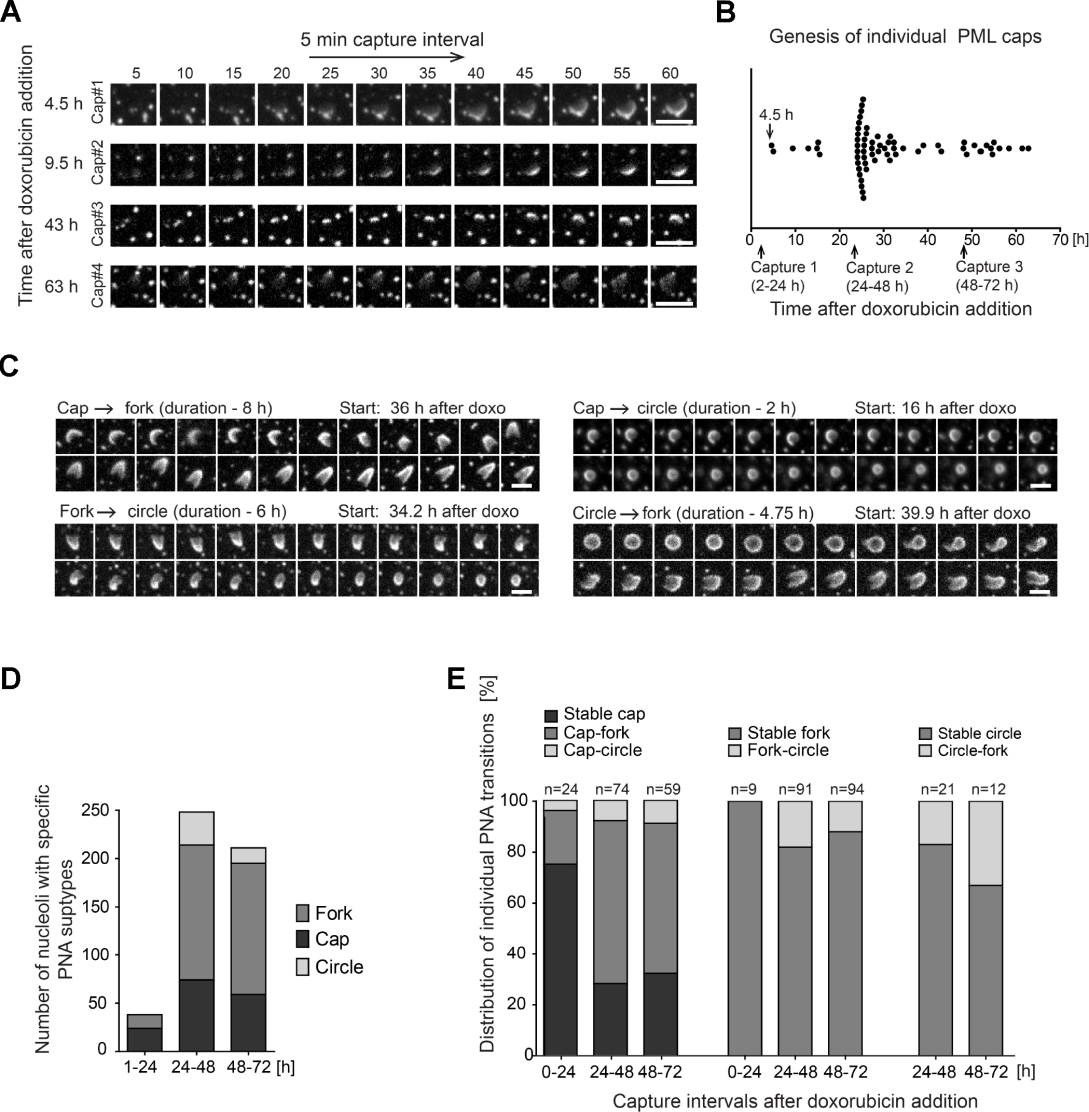
**Structural transitions of PNAs subtypes.** RPE-1^*hTERT*^ stably expressing EGFP-PML IV were analyzed by live-cell imaging in three consecutive capturing sessions spanning 2–24, 24–48 and 48–72 hours after doxorubicin treatment (0.75 μM). (**A**) Series of sequential images of four individual cap formations (cap#1–4) that initiated 4.5, 9.5, 43 and 63 hours after doxorubicin addition. (**B**) Plot of all genesis of PML-caps recorded during three capturing intervals. Dots represent *de novo* formation of individual PML-caps. (**C**) Time-dependent evolution and transition of subtypes of PNAs. The four characteristic transitions – 'cap-to-fork', 'cap-to-circle', 'fork-to-circle' and 'circle-to-fork' – are represented by series of sequential images. The initiation of capturing and the length of recorded time (in parentheses) are given for each type of transition. (**D**) The quantitative distribution of specific subtypes of PNAs in three capturing sessions (see Figure 1B for comparison). (**E**) The quantitative distribution of specific PNAs subtype transitions in three capturing sessions. The PNAs that did not change for over 5 hours were considered as stable. Bars, 4 μm.

To track the genesis of the PNAs formation and to assess whether there is a direct temporal relation/continuum among their individual subtypes we followed the development of the PNAs in RPE-1^h*TERT*^ cells stably expressing the EGFP-PML IV by live-cell imaging after doxorubicin exposure (0.75 μM). In concert with the results showed above in Figure 1C, the cap-like accumulations of otherwise diffuse PML at the nucleolar periphery at 4.5 hours after doxorubicin addition, were the first type of PNAs that appeared (see Figure 4A and complementary Supplementary Videos 4–7). It should be emphasized that new caps emerged in cells continuously during the whole monitoring time (60 hours) after doxorubicin addition, indicating that the signal for the formation of PNAs is not limited to the specific time but present for a long time period. The percentage of cells with the new cap peaked around 28 hours after doxorubicin addition suggesting the period of the highest manifestation of the signal for PNAs formation (Figure 4B).

The caps then gradually evolved into either the fork-like structures or the circles but a reversion between the forks and circles was observed as well (see Figure 4C and complementary Supplementary Videos 8–11). Figure 4D shows quantification of a proportion of individual PNAs during the entire monitored period of 72 hours divided into three intervals: 1–24, 24–48 and 48–72 hours. The total extent of PNAs formation was highest in the 24–48-hour interval consistently with the non-continuous, time-course end-point observations of the PNAs formed by endogenous PML (Figure 1C). The stability and frequency of transitions among specific PNAs during 72 hours of doxorubicin exposure is shown in Figure 4E. The most frequent PNAs transitions observed were cap-to-fork, the less frequent cap-to-circle, fork-to-circle and circle-to-fork. Based on quantitative evaluations it was also evident that most forks and circles were stable over 5 hours.

Altogether, the time-lapse analysis revealed that the formation of PNAs started by the accumulation of the initially diffuse PML on the pole of the nucleolus and the signal for such association persisted over several days after stress initiation. This data also clearly demonstrates that structurally different PNAs are actually temporally distinct, dynamic developmental stages of newly formed PML nucleolar compartments.

### Genesis, stability and fate of late PNAs: PML-NDS

**Figure 5:**
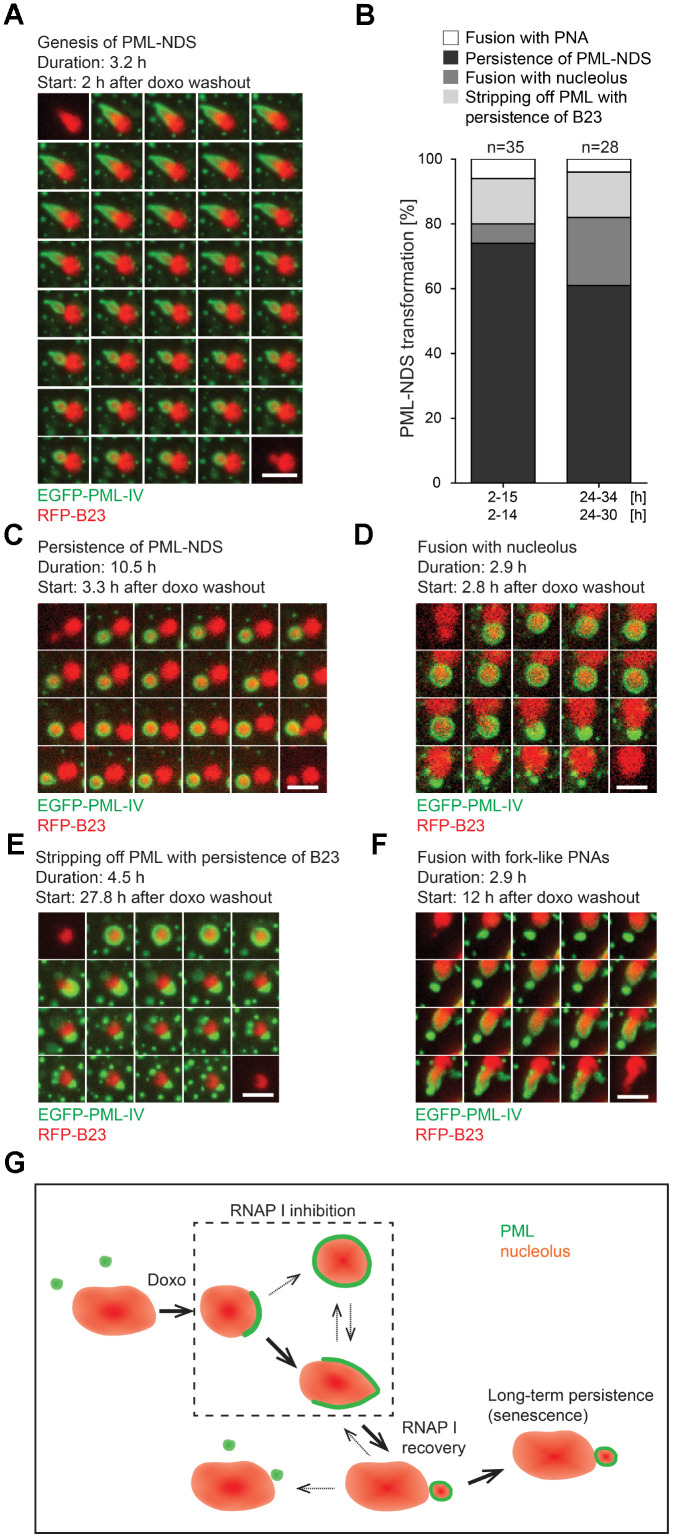
**The genesis, stability and fate of PML-NDS.** RPE-1^*hTERT*^ stably expressing EGFP-PML IV (green) and RFP-B23 (red) were treated with 0.75 μM doxorubicin for 48 hours and analyzed by live-cell imaging after doxorubicin washout (up to 34 hours) for the presence of PML-NDS. (**A**) Series of sequential images mapping the genesis of PML-NDS. (**B**) Comparison of proportional representation of PML-NDS fates between two time-lapse capturing sessions (experiment I: 2–15 and 24–34 hours; experiment II: 2–14 and 24–30 hours) after drug removal. Four different fates of PML-NDS were monitored: persistence (no change) (**C**), fusion with nucleolus (**D**), stripping of PML with B23 persistence (**E**), and fusion with fork-like PNAs (**F**). EGFP-PML IV, green; RFP-B23, red; bars, 4 μm. The initiation of capturing and the length of recorded time are given for each type of transition. (**G**) Schematic representation of PNAs transmutations recorded by time-lapse microscopy. The bold arrows show the main transition pathways observed.

As shown above, PML-NDS represent the specific type of PML nucleolar associations that developed as the final structural subtype after stress initiation, in connection with stress release (drug removal) and rDNA transcription re-initiation. To trace unambiguously the evolution and fate of such PML-NDS structures, the RPE-1^h*TERT*^ cells expressing EGFP-PML IV together with B23-RFP as a nucleoli-specific marker were exposed to doxorubicin for 48 hours and followed by time-lapse imaging divided into two capture sessions of 2–15 and 24–34 hours after the drug removal. As shown in Figure 5A and Supplementary Video 12, PML-NDS originated predominantly from forks by stripping and enclosing a part of nucleolar material from originally segregated nucleolus immediately after the drug removal. The first PML-NDS were detected 3 hours after the drug removal and they continued to appear during the next 12 hours, indicating that the signal for fork transformation into the PML-NDS lasted during the early period of drug washout (Supplementary Figure 7A) concurrently with the recovery of RNAP I activity (Supplementary Figure 3). As documented in Supplementary Figure 7B, the lifetime of PML-NDS was variable, from 0.8 to 13 hours. Note the exact estimation of PML-NDS lifetime was partly limited due to escape of some cells from the visual field or emergence of some PML-NDS close to the end of the capture interval. However, we can conclude that the maximal lifetime of single PML-NDS was at least 13 hours. The destiny of PML-NDS was variable (see Figure 5B for quantitative cumulative data and Supplementary Figure 7B for the fate of individual PML-NDS). Thus, whereas most PML-NDS were quite stable (74 and 61% in 2–15- and 24–34-hour interval, respectively; Figure 5C and Supplementary Video 13), another three, less frequent modes of their fate were recorded: 1) fusion with the nucleolus with simultaneous reduction of PNAs into the residual regular PML nuclear body (6 and 21% in 2–15- and 24–34-hour interval, respectively; Figure 5D and Supplementary Video 14), 2) stripping of PML into PML NBs with persistence of B23 (14% in both intervals; Figure 5E and Supplementary Video 15), and 3) fusion with the fork-like PNAs (6 and 4% in 2–15- and 24–34-hour interval, respectively; Figure 5F and Supplementary Video 16).

Hence, the PML-NDS appeared apparently as the final stage of the transition sequence of cap/fork/PML-NDS (see Figure 5G for scheme). Note, however, that we also detected *de novo* initiation of this complete sequence during the doxorubicin removal phase (Supplementary Figure 7C and Supplementary Video 17). The PNAs transition sequence from the cap to the PML-NDS after doxorubicin removal lasted between 2–6.5 hours; nevertheless, an incomplete PNAs transition sequence could be observed as well.

It should be stressed that PML-NDS were the only PNAs structures present when very low doses of doxorubicin (0.075 and 0.375 μM) were applied (see Supplementary Figure 1A), suggesting that a low-level nucleolar stress may induce them directly, without the preceding stages of the PML nucleolar associations (caps, forks, circles), i.e. in a transient period of inhibition of RNAP I activity and nucleolar segregation. Whether these two forms of PML-NDS are functionally equivalent or not needs to be further elucidated.

To conclude, the formation of PML-NDS was linked to restoration of nucleolar activity by transition from early PML nucleolar associations in a process comprising a separation of a part of nucleolar and nucleoplasmic content into a detached body. This structure can either fuse with nucleolus or persist for a long time, the latter pattern indicating a block or irreversibility of a normal PNAs transition sequence.

### PNAs co-associate with DNA damage foci

**Figure 6:**
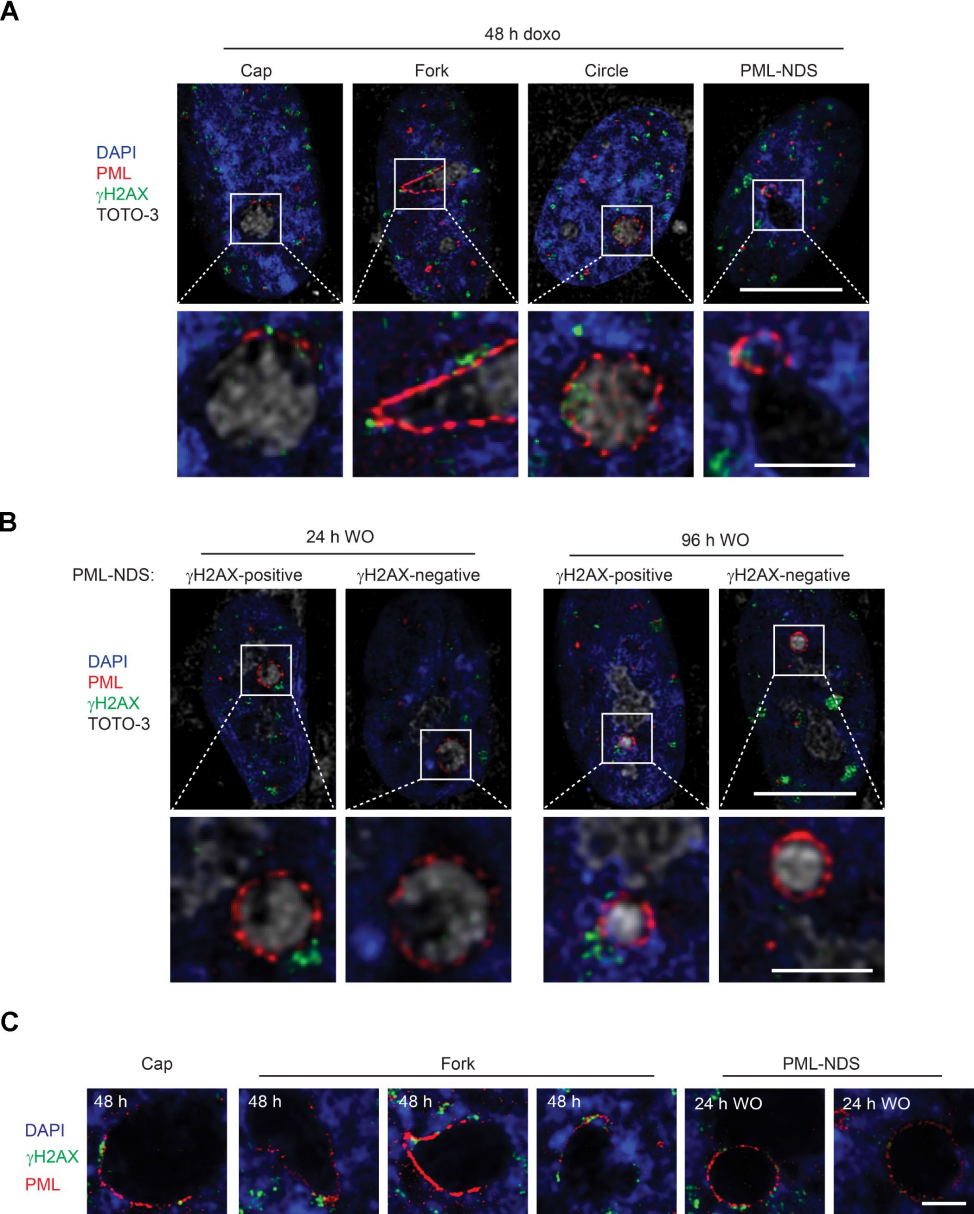
**PNAs colocalize with persistent DNA damage foci.** Co-association of DNA damage foci and PNAs detected by γH2AX (green) and PML (red) immunostaining, respectively, in RPE-1^*hTERT*^ treated with 0.75 μM doxorubicin for 48 hours (**A**) and 24 and 96 hours after drug removal (**B**). Higher magnifications of confocal microscopic images of PML/γH2AX co-associations (insets) are shown in the lower rows. Bars, 10 μM for the whole cells and 3 μM for the insets. (**C**) High resolution STED microscopic images of PNAs/γH2AX co-associations in RPE-1^*hTERT*^ treated with 0.75 μM doxorubicin for 48 hours and followed for PML-NDS appearance 24 hours after drug removal (WO). Nuclei are stained with DAPI (blue). Bars, 4 μM.

As PML nuclear bodies associate with irreparable DNA damage foci [21–24] characteristic for senescent cells [25, 26] and doxorubicin induces DNA damage and DNA damage response (DDR), we analyzed next whether there is an association of the PNAs with a DNA damage marker, histone H2A.X phosphorylated on serine 139 (γH2AX; [35]). Using confocal fluorescence microscopy, we found that 48 hours after doxorubicin addition all types of PNAs (caps, forks, circles and PML-NDS) associated with γH2AX signal (Figure 6A). Furthermore, to examine whether all the PML-NDS stay in contact with the DNA lesions we compared the cells 48 hours after addition of doxorubicin with those 24 and 96 hours after doxorubicin washout. We found out that all PML-NDS captured before doxorubicin washout (i.e. 48 hours after doxorubicin addition) associated with γH2AX, which was very similar to the sample obtained 24 hours after doxorubicin washout (the γH2AX signal was not detected only in one PML-NDS from 13 captured). However, 96 hours after doxorubicin washout only 60% of analyzed PML-NDS (n = 14) contained detectable γH2AX signal (Figure 6B).

The association of early PNAs with γH2AX was further assessed by super-resolution microscopy. Using the STED microscopy, a clear co-association of the PML with γH2AX on the nucleolar border was detected (Figure 6C). Intensity profiling of STED images of cap-, fork-, circle-like and PML-NDS forms of PNAs (Supplementary Figure 8) showed both overlapping and flanking patterns of both signals, the latter indicating similar juxtaposition of the PML to the DNA damage foci as described for the nuclear PML NBs/DNA damage lesions [25].

To conclude, the PNAs formed and localized closely with the DNA damage lesions at the nucleolar periphery in cells exposed to doxorubicin. These findings indicate that PML can be involved in the repair of lesions formed either in rDNA or DNA in the close proximity to rDNA arrays. The persistence of DNA damage response close to nucleoli suggests that the damage of nucleolus-associated DNA can contribute to the pool of irreparable DNA lesions responsible for senescence induction.

## DISCUSSION

Cellular senescence emerges as an important factor contributing to aging and aging-associated diseases (for a review, see ref. [2]). Mechanistically, cellular senescence is a long-lasting cell cycle arrest due to prolonged activity of cell cycle checkpoints. DNA damage is considered as a key upstream activator of checkpoint response of senescent cells, however, the nature of the DNA “irreparability” or DDR signaling persistency has not been fully resolved yet. The elucidation of the causal mechanisms of cellular senescence might facilitate a discovery of new approaches to control aging.

The interaction of PML with nucleolar components was reported as a characteristic feature of replicative senescence of human mesenchymal stem cells [29]. In our present study, as a basic prerequisite for understanding the role of the PML-nucleolar subcompartment in development of cellular senescence, we analyzed the genesis and dynamics of PML-nucleolar structures in the context of premature senescence induced by the chemotherapeutic drug doxorubicin. Employing live-cell imaging time-lapse and high-resolution microscopy, we have found that 1) the dynamic changes of PML-nucleolar associations are intimately linked to inactivation and reactivation of RNAP I transcription, respectively; 2) the PML-nucleolar compartment develops dynamically in a sequence of events that includes structurally distinct, yet temporally linked PNAs, eventually leading to either their resolution or persistence; 3) all the PNA subtypes associate with the markers of DNA damage; 4) the long-lasting PML nucleolar structures (PML-NDS) also commonly associate with markers of DNA damage, thereby implicating the PNAs in persistent DDR typical of senescent cells; and 5) the formation of such PNAs depends on intact PML.

We provide evidence that the PML nucleolar compartment undergoes continuous structural reorganizations associated with the repression of RNA polymerase I activity. It was reported that after RNAP I inhibition by actinomycin D or after rDNA damage, several proteins including subunits of RNAP I and also rDNA relocate from nucleolar interior to the nucleolar periphery forming the so-called nucleolar cap [34, 36, 37]. Importantly, we revealed an intimate relationship between the ‘cap’ and ‘fork’ subtypes of PNAs with the canonical nucleolar caps (i.e. those formed by segregated PAF49), where the PNAs form structural shields/covers juxtaposed to nucleolar caps, a scenario suggesting that the cap-specific factors could interact with the PML. Nevertheless, despite all nucleoli were segregated at the specified time, only fraction of them formed the PNAs. This indicates that individual nucleoli could be in different functional states, and that the nucleolar segregation itself is not sufficient to provide the signal for accumulation of PML at the nucleolar surface, a process that seems to require an additional factor(s).

The dynamic nature of the structural reorganization of PNAs reported here raises a question about forces governing the process. The PML nuclear bodies and nucleoli are membrane-less compartments and it is postulated that the chemico-physical nature of formation of these structures is a liquid-liquid phase separation ([12], for reviews, see refs. [38, 39]). It should be emphasized that the PML interacts with the nucleolus only under specific stress conditions. Ectopic (over)expression of the PML isoforms was insufficient to establish PML interaction with the nucleoli (our unpublished data) indicating that this interaction was not a result of a saturation of the PML NB compartment. Moreover, the formation of asymmetric funnel-like shape PNAs can be hardly accounted for by liquid-liquid phase separation as a sole mechanism of its genesis. Although the forces behind the transition sequence of PNAs are unknown, several mechanisms including the asymmetric flux of ribosome biogenesis components linked to the asymmetry of segregated nucleolus, perinucleolar chromatin movement or an involvement of mechanical forces of nucleolar motors during the DNA damage response and repair (see further) can be assumed.

Arguably the most intriguing feature of the PNAs was their intimate and long-term co-association with markers of the DNA damage. Even the early-stage PNAs were positive for phosphorylated histone H2AX. From the dynamics of PNAs and the presence of DNA damage foci in a fraction of the persistent PML-NDS we assume that during the cap/fork/PML-NDS transition sequence the PML concentrates the damaged DNA originally spread on nucleolar surface to one pole of the nucleolus, the tip of the funnel-like structure, which is then further detached from the functionally restored nucleoli into a separated structure, PML-NDS. Such sequestration of damaged DNA could serve as a mechanism protecting the interference of ongoing DNA repair with reestablished nucleolar transcription. Notably, recent evidence indicates that β-actin and nuclear myosin I are the factors necessary for repositioning of RNAP I and rDNA back into nucleolar interior after successful completion of rDNA repair [40]. Whether also the structural transitions of PNAs are the results of similar mechanical forces should be explored.

The last stage of PNAs, the PML-NDS, emerges almost exclusively from the tip of the funnel-shaped PNAs during stress release (here drug removal). The presence of highly concentrated proteins such as DHX9 and B23 in these structures indicates ongoing active sequestration of nucleolar factors into this structure. The force behind the genesis of PML-NDS might be a tendency of PML to form a balloon-like structure with the lowest energy state like in the PML NBs. Alternatively, the transmutation of funnel-like PNAs into PML-NDS may be a consequence of the transition of segregated nucleolus into the active one. Reactivation of nucleoli is accompanied by volume growth and reorganization of the nucleolar interior that may cause an alteration of nucleolar surface weakening the interactions essential for maintenance of the PNAs. However, the external (mechanical) forces cannot be excluded either.

The further fate of the DNA segregated in PML-NDS may be multiple, including its degradation or repair, consistent with the notion we present here, namely that only a fraction of PML-NDS remains positive for phosphorylated histone H2AX at later time-points after doxorubicin washout.

We observed that a significant subset of PML-NDS decays by three distinct mechanisms which, however, could be functionally equivalent. We propose that a loss of phosphorylated histone H2AX from the PML-NDS or a disappearance of the whole PML-NDS reflect a successful DNA repair event, whereas those PML-NDS remaining persistently positive for phosphorylated histone H2AX likely highlight persistence of unrepaired rDNA lesions.

Notably, some PML-NDS can persist for very long time periods (at least 17 days after doxorubicin washout). This is further underscored by the presence of PML-NDS in senescent cells [29], the co-association of PML NBs with persistent DNA lesions [26], and the recent evidence that an introduction of DNA double strand breaks into rDNA loci results in premature senescence [10], all indicating that nucleoli-associated chromatin is, besides telomeric loci [6, 7, 41], another type of DNA locus sensitive to specific senescence-inducing genomic damage.

The exact function of PML in the DNA repair is still under investigation. Recent evidence points to the involvement of PML and PML nuclear bodies in homologous recombination-directed DNA repair (HDR; [25, 28]). Boichuk *et al.* showed that knockdown of PML affected HDR efficiency, possibly reflecting an inability to accumulate several HDR proteins (Rad51, Mre11 and BRCA1) in DNA lesions [28]. Recently, Vancurova *et al.* reported that PML knockout cells revealed higher sensitivity to treatments causing DNA lesions requiring repair by HDR [25]. Both findings indicate that PML may modulate the HDR and thus the cell fate under genotoxic stress. As shown recently, proteins of both pathways (NHEJ and HR) colocalized with DSB presented in rDNA [36, 37, 42]. Warmerdam *et al.* implied that HR-directed repair can block the repair of rDNA breaks inserted by I-PpoI or CRISPR/Cas9 and when HR is involved in repair of such rDNA breaks the rDNA copy number is significantly reduced [42]. In general, HDR is considered less erroneous compared to NHEJ [43], however, in case of rDNA, there is a substantial risk of recombination event occurring between two rDNA repeats in *trans* or *cis*, resulting in extensive translocations and deletions, both leading to genomic instability [42]. Nevertheless, the several variants of HDR pathways were described [44, 45] and some of them may specifically participate in rDNA maintenance by proper repair of rDNA, as was proposed recently [46, 47]. One of the regulators of HDR is BLM helicase [48–51], depletion of which destabilized rDNA [52]. Notably, our results indicate that BLM accumulated in PNAs (see Supplementary Figure 9) implying that PNAs may modulate the local concentration of proteins involved in DDR and thus contribute to regulation of HDR. Altogether, it can be proposed that the PNAs are involved in a specific form of DNA repair, likely of difficult-to-repair DNA breaks in damaged rDNA repeats. Although a direct evidence that PML associates with damaged rDNA loci was not provided, the findings that the damage of ribosomal DNA is linked to relocation of rDNA to nucleolar caps [36, 37, 53] underscores such notion.

In conclusion, the interaction of PML with the nucleolus is a dynamic process linked to inactivation of RNA polymerase I, with the ensuing nucleolar segregation in response to stress. The association of PML nucleolar compartment with the rDNA damage lesions implicates the dynamic PNA structures reported here in DNA repair and separation of unrepaired DNA from nucleoli especially during the period of subsiding stress and reconstitution of nucleolar function. Whether senescence-inducing insults other than those examined here, such as oncogene-induced stresses that are associated with enhanced PML bodies accumulation [54] and DNA breakage [55] also lead to formation of PML-NDS reminiscent of those we describe in this study, remains to be elucidated.

## MATERIALS AND METHODS

### Chemicals and antibodies

4′,6-diamidino-2-phenylindole (D9542), doxorubicin hydrochloride (D1515), 5-fluorouridine (F5130), G418 disulfate salt (G5013) and puromycin dihydrochloride (P7255) were obtained from Sigma-Aldrich/Merck (Darmstadt, Germany); Click-iT EdU Alexa Fluor 488 Imaging Kit (C10337) and TOTO-3 (T-3604) were obtained from Thermo Fisher Scientific (Waltham, MA, USA); SiR-DNA dye (SC007) was purchased from Spirochrome (Stein am Rhein, Switzerland); Antifade Pro-long Gold Mounting Media (AF-400-H) was obtained from Immunological Sciences, Rome, Italy). Specification of primary and secondary antibodies used throughout the study is listed in Supplementary Table 1.

### Cell culture

Immortalized human retinal pigment epithelial cells (RPE-1^*hTERT*^, ATCC), RPE-1^*hTERT*^ PML knockout cells [25], RPE-1^*hTERT*^ cells stably expressing EGFP-PML IV or EGFP-PML IV together with RFP-B23, and primary human diploid fibroblasts (BJ, ATCC), all checked for absence of mycoplasma infection, were cultured in Dulbecco’s modified Eagle's medium (Gibco/Thermo Fisher Scientific, Waltham, MA, USA) containing 1.5 g/L (BJ) or 4.5 g/L (RPE-1) glucose and supplemented with 10% fetal bovine serum (Gibco/Thermo Fisher Scientific, Waltham, MA, USA) and antibiotics (100 U/mL penicillin and 100 μg/mL streptomycin sulfate, Sigma, St. Louis, MO, USA). Primary human mesenchymal stem cells (hMSC, ATCC) were grown in mesenchymal stem cells growth medium (composed of medium PT 3238 and supplements PT 4105, Lonza, Basel, Switzerland). The cells were cultivated in normal atmospheric air containing 5% CO_2_ in a standard humidified incubator at 37°C, on a tissue culture dish (TPP Techno Plastic Products AG, Trasadingen, Switzerland). Doxorubicin was used at final concentrations 0.75, 0.375 or 0.075 μM.

### Plasmid construction and lentiviral transduction

For stable expression of PML isoform IV tagged with EGFP at N-terminus, lentiviral vector pCDH-EGFP-PML IV was prepared as follows: PCR-amplified PML IV cDNA was inserted into *Hinc*II-digested pGEM4z plasmid (P2161, Promega, Madison, WI, USA) and PML IV was subsequently excised and inserted into pEGFP-C3 plasmid (6082-1, Clontech/TaKaRa, Kusatsu, Shiga, Japan) via the *Hind*III/*BamH*I sites. Finally, EGFP-PML IV was excised and inserted into the *Bmt*I/*BamH*I sites of pCDH-CMV-MCS-EF1-Puro vector (CD510B-1, System Biosciences, Palo Alto, CA, USA). For stable expression of B23 tagged with RFP at N-terminus, lentiviral vector pCDH-RFP-B23 was prepared as follows: RFP-B23 was excised by *BamH*I/*Nhe*I from the mRFP-C2-B23 plasmid that was kindly provided by A. Holoubek [56]. The fragment was inserted into pCDH-CMV-MCS-EF1-Neo vector (CD514B-1, System Biosciences, Palo Alto, CA, USA), digested by the same restriction enzymes. The stable RPE-1^*hTERT*^ cell lines expressing EGFP-PML IV and RFP-B23 were generated by lentiviral infection using the respective pCDH-CMV-MCS-EF1 vectors and subsequent selection with puromycin (15 μg/mL) and neomycin (1.12 mg/mL). Afterwards, the cells were sorted for low expression of the fluorescence markers using a cell sorter (BD Influx Cell Sorter, BD Biosciences, San Jose, CA, USA).

### Indirect immunofluorescence, confocal microscopy, ScanR microscopy and stimulated emission depletion (STED) microscopy

Cells grown on glass coverslips were fixed with 4% formaldehyde in PBS for 15 min, permeabilized in 0.2% Triton X-100 in PBS for 10 min, blocked in 10% FBS in PBS for 30 min, and incubated with primary antibodies for 1 hour, all in RT. In case of BLM staining, pre-extraction with a specific pre-extraction buffer (0.5% Triton X-100, 20 mM HEPES pH 7.4, 50 mM NaCl, 3 mM MgCl_2_, 1 mM PMSF, 10 mM β-glycerol phosphate) for 10 min was done before cell fixation. After that, cells were washed three times 5 min in PBS, and secondary antibodies (Alexa Fluor 555 used specifically for STED) were applied in RT for 1 hour. For some experiments, TOTO-3 was applied together with secondary antibodies. Subsequently, cells were counterstained with 1 μg/mL DAPI for 2 min, washed 3 times with PBS for 5 min, let dry and mounted with Antifade Pro-long Gold Mounting Media. The wide-field images were subsequently acquired on the Leica DM6000 fluorescent microscope using the HCX PL APO 63×/1.40 OIL PH3 CS and HCX PL APO 40×/0.75 DRY PH2 objectives and monochromatic CCD camera Leica DFC 350FX (Leica Microsystems GmbH, Wetzlar, Germany); the confocal images were acquired on microscope DMI6000 with laser scanning confocal head Leica TCS SP5 AOBS Tandem, using the HC PL APO 63×/1.40 OIL CS2 objective (Leica Microsystems GmbH, Wetzlar, Germany). High-content image acquisition was done on the Olympus IX81 microscope (Olympus Corporation, Tokyo, Japan) equipped with ScanR module using the UPLFN 40×/1.3 OIL objective and sCMOS camera Hamamatsu ORCA-Flash4.0 V2 (Hamamatsu Photonics, Shizuoka, Japan). The data were analyzed in ScanR Analysis software (Olympus Corporation, Tokyo, Japan). The DHX9 accumulations and γH2AX foci were detected by the Spot detector function. The percentage of cells containing 3–15 γH2AX foci was counted from EdU-negative cells only, since untreated S-phase cells often exhibit high γH2AX signal and would therefore perturb the analysis. For super-resolution STED microscopy, the cells were mounted with glycine/N-propyl gallate and imaged on the microscope DMi8 with laser scanning confocal head Leica TCS SP8 and STED 3X module using the HC PL APO100×/1.40 OIL STED WHITE CS2l objective. Image deconvolution of confocal and STED images was done using Huygens Professional software (Scientific Volume Imaging B.V., Hilversum, The Netherlands).

### Live-cell structured illumination microscopy (SIM) and time-lapse microscopy

RPE-1^*hTERT*^ cells stably expressing EGFP-PML IV or EGFP-PML IV and RFP-B23 were seeded on glass-bottom plastic dishes (HBST-3522, WillCo Wells, Amsterdam, The Netherlands) and cultivated in FluoroBrite™ DMEM (A1896701, Gibco/Thermo Fisher Scientific, Waltham, MA, USA) supplemented with 10% FBS. This medium was also used instead of classical DMEM for all indicated doxorubicin treatments and removals. For super-resolution SIM microscopy, 1 μM SiR-DNA dye was added to the cells at least 1 hour before capturing and the live cells were then imaged on the DeltaVision OMX™ V4 imaging system with the Blaze SIM Module (Applied Precision/GE Healthcare, Chicago, IL, USA), using the PLAN APO N 60×/1.42 OIL objective (Olympus Corporation, Tokyo, Japan) and pco.edge 5.5 sCMOS cameras (PCO AG, Kelheim, Germany). Image reconstruction and registration was done in softWoRx software (Applied Precision/GE Healthcare, Chicago, IL, USA). For time-lapse microscopy, the live cells were imaged on the inverted fluorescence Olympus microscope version IX-71 (Olympus Corporation, Tokyo, Japan) coupled with the Delta Vision Core system (GE Healthcare, Chicago, IL, USA), using the APO/340 40×/1.35-0.65 CORR OIL objective (Olympus Corporation, Tokyo, Japan) and CoolSANP HQ CCD camera (Teledyne Photometrics, Tucson, AZ, USA). All live-cell imaging was done at 37°C in normal atmospheric air containing 5% CO_2_. Tracking of selected cells was done post-imaging in ImageJ, using the Resolve3D plugin.

### 5-Fluorouridine incorporation assay

Cells were incubated with 1 mM 5-fluorouridine (5-FUrd) for 30 min, at indicated time-points after doxorubicin treatment and removal. After that, cells were fixed with 4% formaldehyde at RT for 15 min and the 5-FUrd incorporation was visualized using anti-BrdU antibody cross-reacting with 5-FUrd. The standard protocol for immunofluorescence described above was used.

### Cell proliferation assay

Cells were incubated with 10 μM 5-ethynyl-2´-deoxyuridine (EdU) for 6 hours and fixed with 4% formaldehyde at RT for 15 min at indicated time-points. To visualize EdU incorporation, click chemistry was performed with Click-iT EdU Alexa Fluor 488 Imaging Kit according to manufacturer’s instructions.

### Senescence-associated beta-galactosidase assay

Cells were fixed with 0.5% glutaraldehyde at RT for 15 min at indicated time-points. After that, cells were washed twice with 1 mM MgCl_2_/PBS and incubated with X-Gal staining solution for 3 hours at 37°C. The staining was terminated by three consecutive washes with ddH_2_O. Finally, the cells were let dry, mounted with Antifade Pro-long Gold Mounting Media and imaged on the Leica DM6000 fluorescent microscope using the HC PLAN APO 20×/0.70 DRY PH2 objective and color CCD camera Leica DFC490 (Leica Microsystems GmbH, Wetzlar, Germany).

### SDS-PAGE and immunoblotting

Cells were harvested into Laemmli SDS sample lysis buffer (62.5 mM Tris-HCl, pH 6.8, 2% SDS, 10% glycerol), boiled at 95°C for 5 min, sonicated and centrifuged at 18,000 × g for 10 min. Concentration of proteins was estimated by the BCA method (Pierce Biotechnology Inc., Rockford, USA). Equal amounts of total protein were mixed with DTT and bromphenol blue to final concentration 100 mM and 0.01%, respectively, and separated by SDS-PAGE (8% or 12% polyacrylamide gels were used). The proteins were electrotransferred to a nitrocellulose membrane using wet transfer. Immunostaining followed by ECL detection was performed. The intensity of DHX9 bands was measured in ImageJ Gel Analyzer plugin. The DHX9 level was calculated as the intensity of DHX9 bands related to the intensity of loading control, while the relative intensity of untreated PML-WT cells was set as one.

## Supplementary Material

Supplementary Figures

Supplementary Table 1

Supplementary Video 1

Supplementary Video 2

Supplementary Video 3

Supplementary Video 4

Supplementary Video 5

Supplementary Video 6

Supplementary Video 7

Supplementary Video 8

Supplementary Video 9

Supplementary Video 10

Supplementary Video 11

Supplementary Video 12

Supplementary Video 13

Supplementary Video 14

Supplementary Video 15

Supplementary Video 16

Supplementary Video 17
