# Supplementary material for: Dynamic PML protein nucleolar associations with persistent DNA damage lesions in response to nucleolar stress and senescence-inducing stimuli
Source: Aging (Albany NY). 2019 Sep 7;11(17):7206–35. doi: 10.18632/aging.102248 (PMC6756913; doi:10.18632/aging.102248)
Supplement: Supplementary Figures [file aging-11-102248-s001.pdf]

SUPPLEMENTARY FIGURES

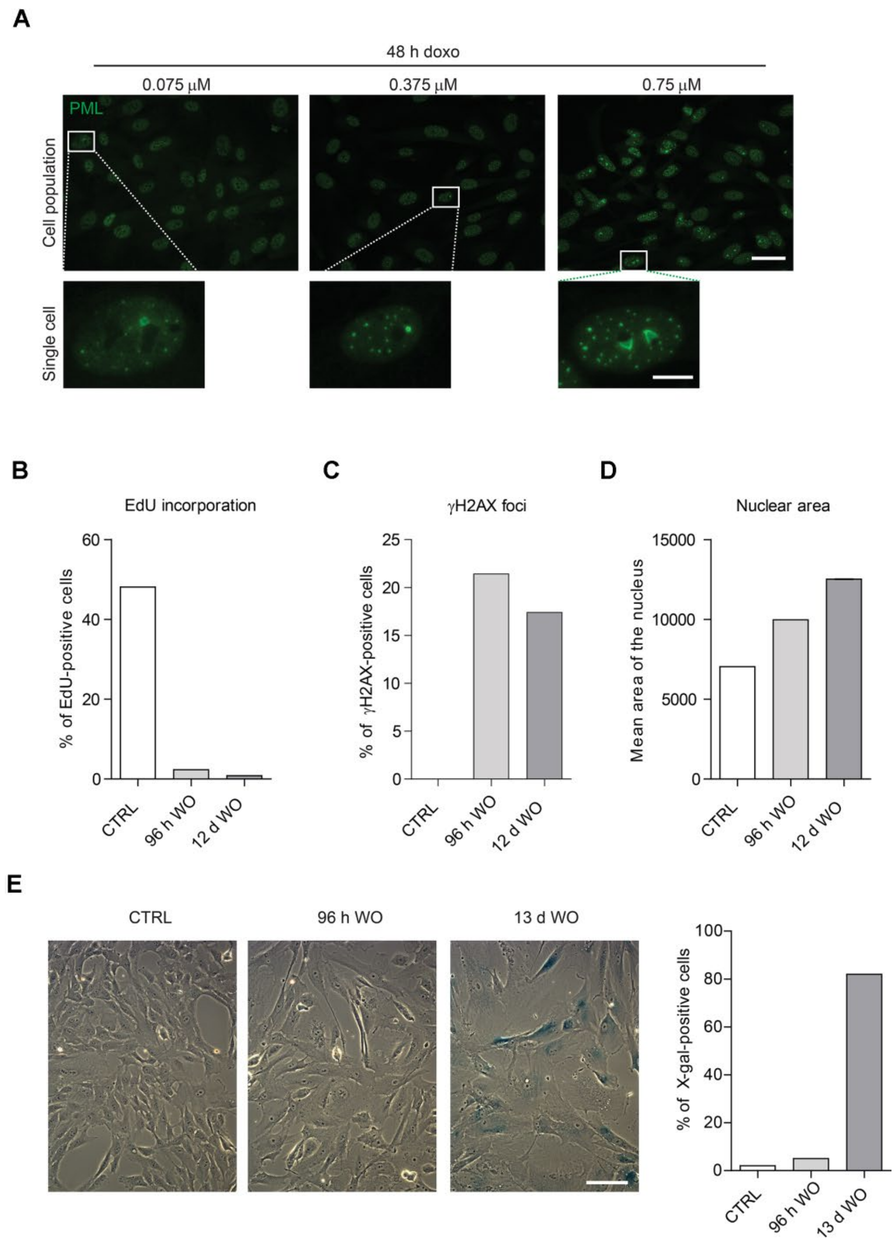

Supplementary Figure 1. The dose-dependent formation of PML nucleolar associations (PNAs) and doxorubicin-induced senescence. (**A**) RPE-1^{hTERT} were treated with 0.075 µM, 0.375 µM and 0.75 µM doxorubicin for 48 hours and PNAs were detected by PML indirect immunofluorescence. Wide-field immunofluorescence images of cell populations (upper row; bar, 50 µm) and individual cells (lower row; bar, 10 µm) are shown. The images were captured with 40×/0.75 objective. (**B**–**D**) RPE-1^{hTERT} were treated with 0.75 µM doxorubicin for 48 hours, after that the drug was removed and the cells were further cultured. At two time-points after drug removal (96 h WO, 12 d WO), the cells were incubated with EdU for 6 hours and stained for γH2AX foci. ScanR microscopic images were analyzed for percentage of EdU-positive cells (**B**), percentage of cells with 3–15 γH2AX foci (**C**), and the mean area of the nucleus (**D**). (**E**) RPE-1^{hTERT} were treated as in (**B**–**D**). Senescence-associated β-galactosidase activity was estimated in untreated and doxorubicin-treated cells at two time-points after drug removal (96 h WO, 13 d WO) and the cells were imaged with 20×/0.70 objective and color CCD camera; bar, 100 µm. To estimate the percentage of β-galactosidase-positive cells, 178, 128 and 92 cells were counted for control, 96 h WO and 13 d WO samples, respectively. (**B**–**E**) The charts present the data from one experiment.

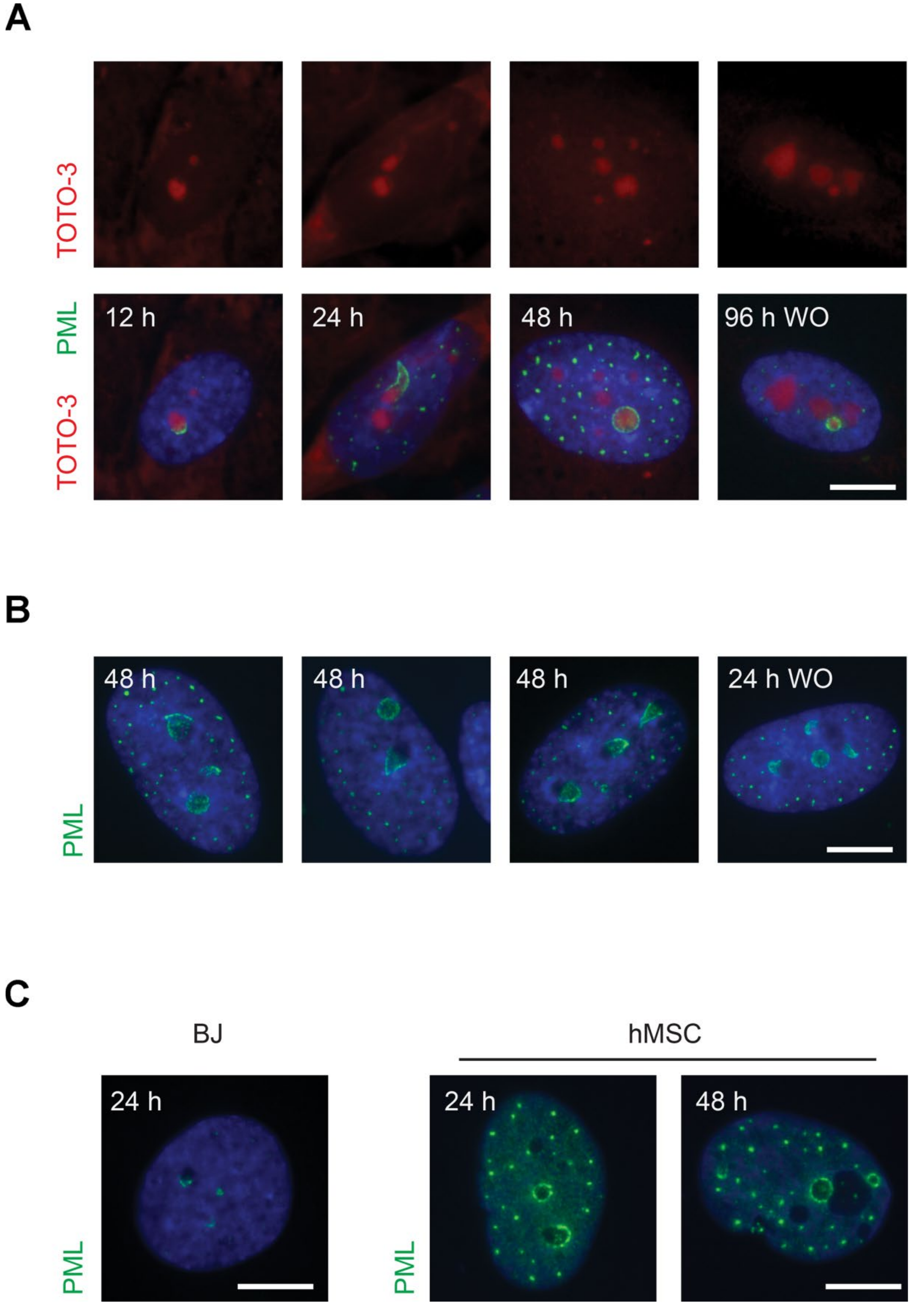

Supplementary Figure 2. The diversity of PML nucleolar associations (PNAs) and their occurrence in different cell types. PNAs detection by PML immunostaining (green) in RPE-1^{hTERT} treated with 0.75 µM doxorubicin for 48 hours and after drug removal (up to 96 hours). Wide-field immunofluorescence images show representative cells, in which nucleoli with and without PNAs (**A**) or with different types of PNAs (**B**) occur at the same time. (**C**) The immunofluorescence detection of PNAs by PML immunostaining in human BJ fibroblasts and mesenchymal stem cells (hMSC) exposed to 0.75 µM doxorubicin. Nuclei and nucleoli were stained with DAPI (blue) and TOTO-3 (red), respectively. The images were captured with 63×/1.4 objective. Bar, 10 µm.

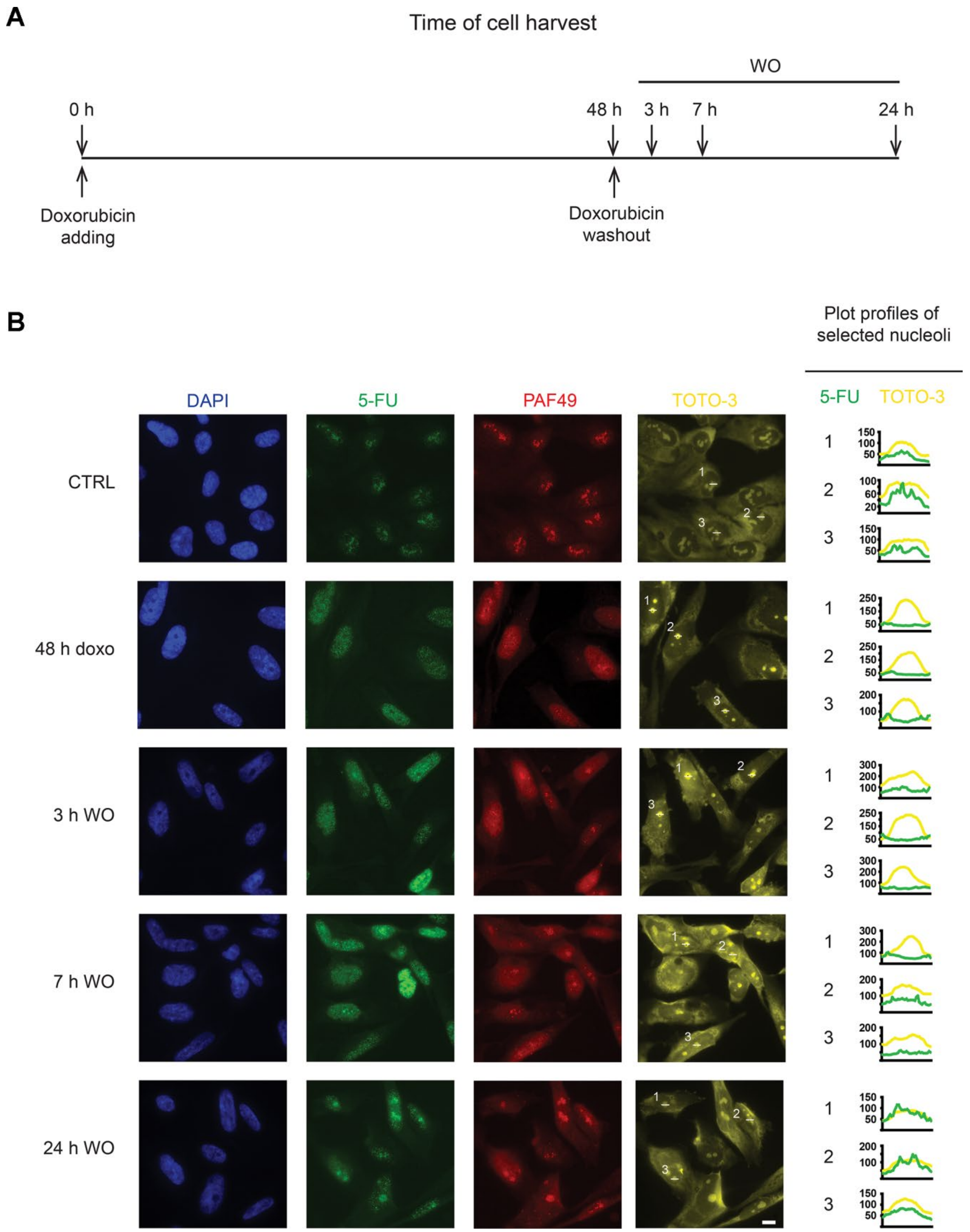

Supplementary Figure 3. RNAP I transcription in the nucleolus is gradually restored during 24 hours after doxorubicin removal. As shown in the scheme of the experiment (**A**), RPE-1^{hTERT} were treated with 0.75 µM doxorubicin for 48 hours; after that doxorubicin was removed and cells were further cultured. Transcription in the nucleolus was estimated by incorporation of 5-fluorouridine (5-FUrd) at indicated time-points. (**B**) The immunofluorescence detection of 5-FUrd (green), representing newly synthesized nucleolar RNA, and localization/segregation of RNAPI subunit PAF49 (red) was performed. The nuclei and nucleoli were stained with DAPI (blue) and TOTO-3 (yellow), respectively. The images were captured with 40x/0.75 objective. Bar, 10 µm. For selected nucleoli, plot profiles of TOTO-3 and 5-FUrd signal were generated, to clearly demonstrate the absence of 5-FUrd signal in inactive nucleoli.

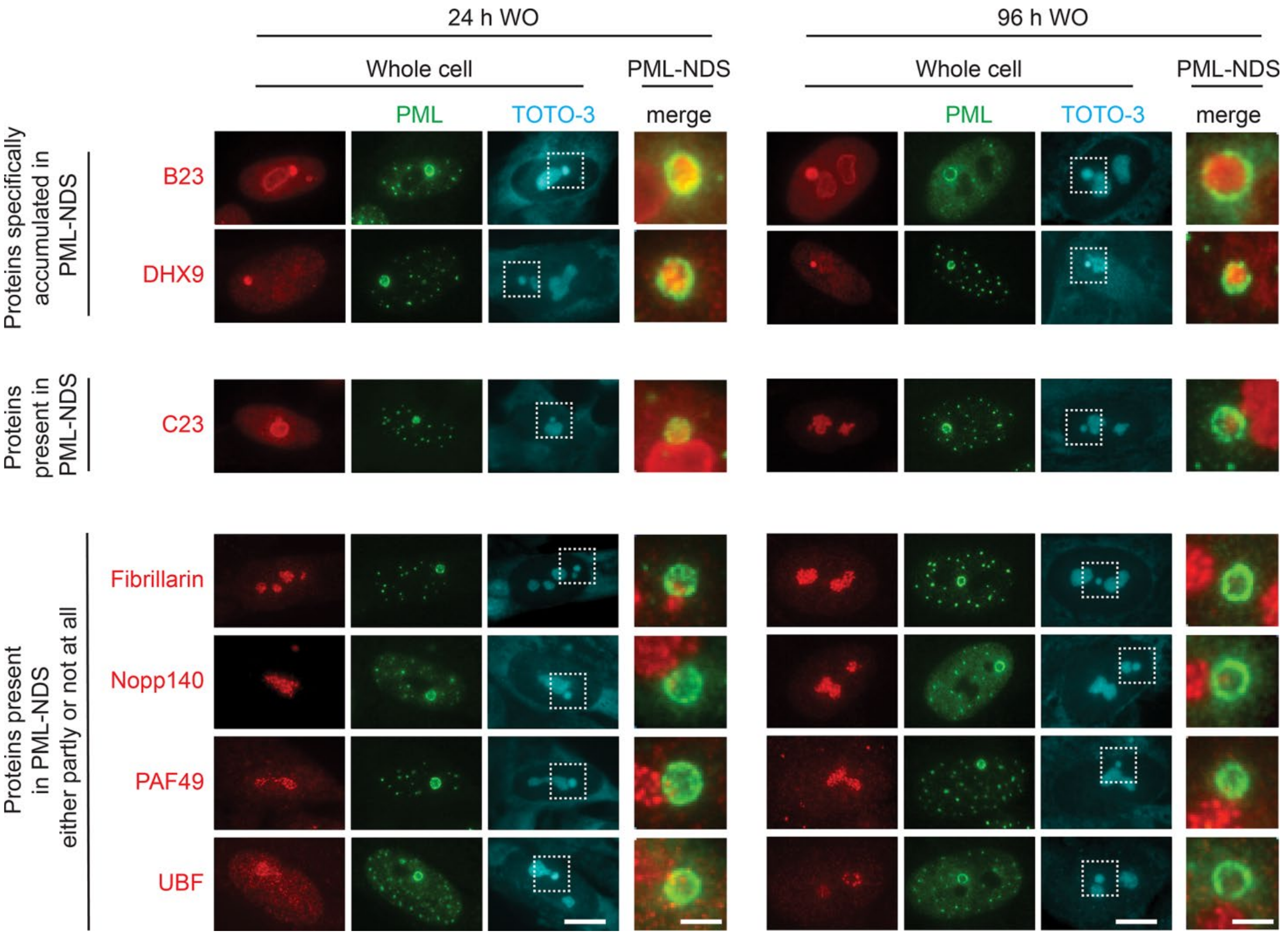

Supplementary Figure 4. Accumulation of proteins into PML-NDS is a selective process. RPE-1^{hTERT} were treated with 0.75 µM doxorubicin for 48 hours; after that doxorubicin was removed and cells were further cultured for 24 or 96 hours. Accumulation of selected nucleolar proteins within PML-NDS was examined by immunostaining with specific antibodies (the proteins of interest – red, PML – green). The nucleoli were visualized by TOTO-3 (cyan). The images were captured with 63x/1.4 objective. Bars, 10 µm (whole cells), 3 µm (insets).

A

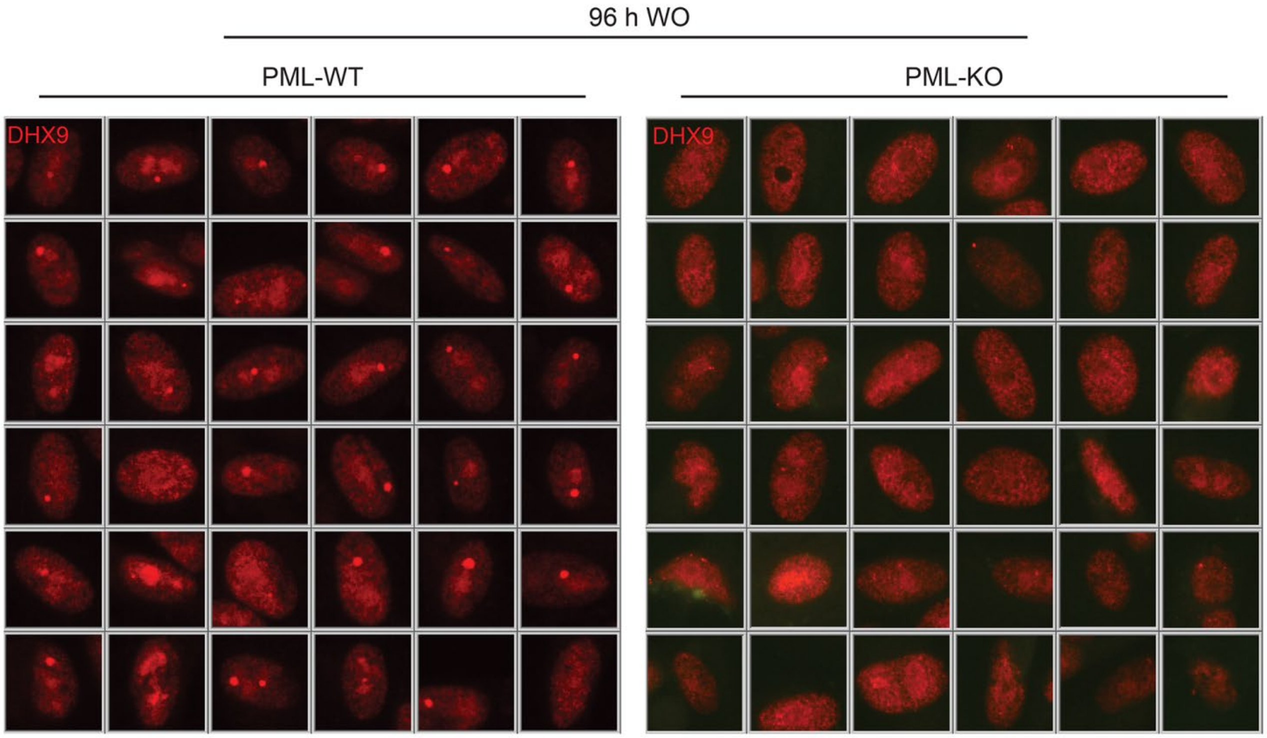

B

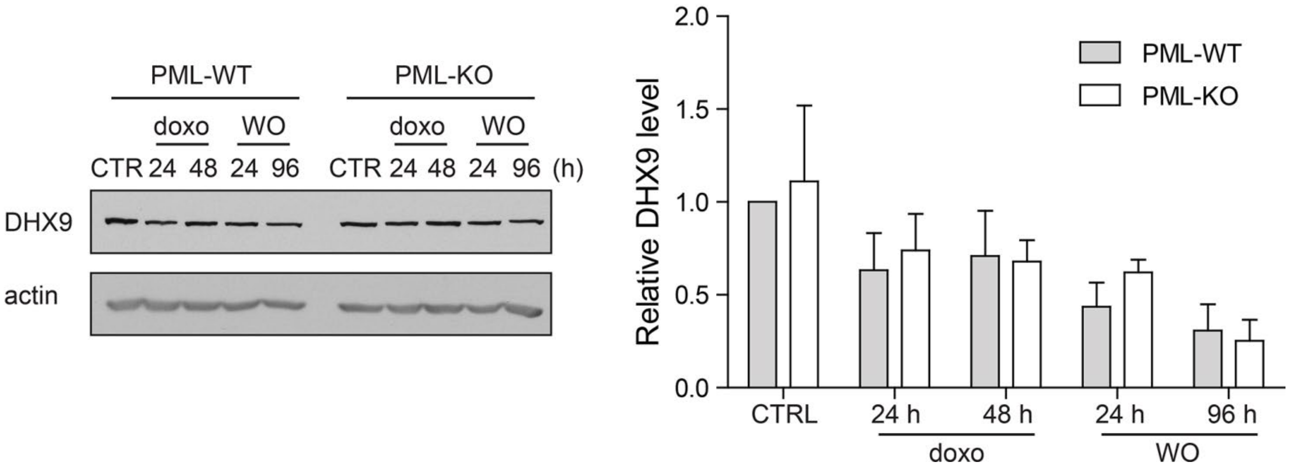

Supplementary Figure 5. Cells identified as positive for DHX9 accumulations and total levels of DHX9 after doxorubicin. RPE-1^{hTERT} PML-WT and PML-KO cells were treated with 0.75 µM doxorubicin for 48 hours; after that doxorubicin was removed and cells were further cultured. (**A**) 96 hours after doxorubicin washout the cells were fixed, stained with antibodies against DHX9 (red) and PML (not shown) and imaged with the ScanR microscope. The galleries of cells identified as positive for DHX9 accumulations were generated as part of the ScanR analysis. Here, thirty-six cells from each gallery are shown. (**B**) At selected time-points during doxorubicin treatment and washout, cell lysates were harvested for SDS-PAGE and immunoblotting (left panel). The DHX9 level was then calculated as the intensity of DHX9 bands related to the intensity of a loading control, while the relative intensity of untreated PML-WT cells was set as one (right panel). Mean values and standard errors of means from three independent experiments are given.

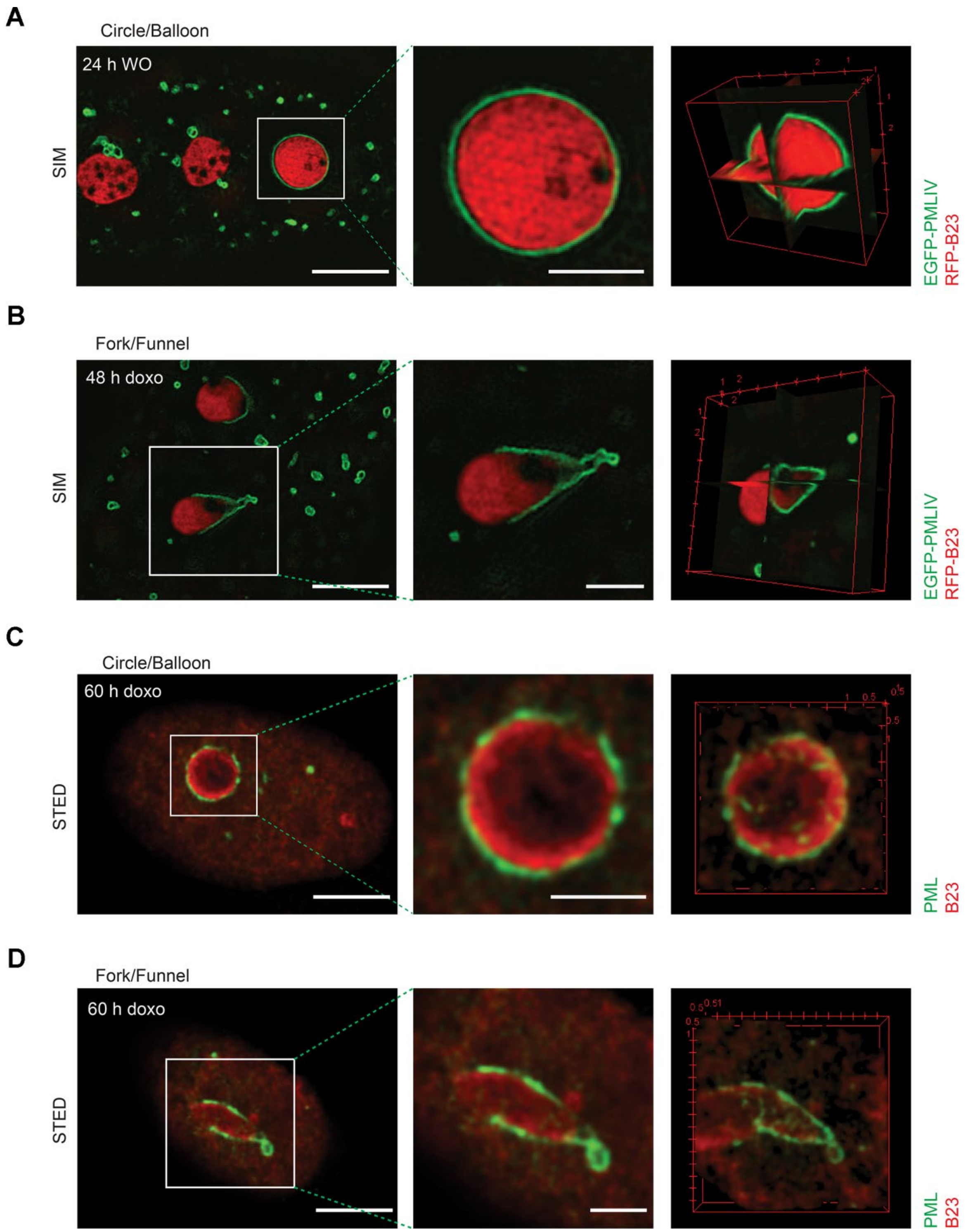

Supplementary Figure 6. PNAs are hollow shells enclosing nucleolar material. PML circles and forks were imaged with two different high resolution microscopy techniques: structured illumination microscopy (SIM) of live doxorubicin-treated RPE-1^{hTERT} cells stably expressing EGFP-PML IV and RFP-B23 (**A**, **B**); and stimulated emission depletion (STED) of fixed doxorubicin-treated RPE-1^{hTERT} cells with endogenous PML and B23 stained by antibodies (**C**, **D**). Central layers of whole cells (left images; bar, 5 μm) and respective PNAs (middle images; bar, 2 μm) are displayed together with 3-D-reconstructed PNAs (right images, for SIM displayed as ortho-views, for STED displayed as whole surfaces). The 3-D reconstruction was done from 22 (**A**), 24 (**B**), 15 (**C**) and 21 (**D**) layers in ImageJ program, using the ImageJ 3D viewer plugin. The distance between two layers is 0.125 μm for SIM and 0.05 μm for STED images; i.e. the depths of the stacks are, namely: 2.75 μm (**A**), 3 μm (**B**), 0.75 μm (**C**) and 1.05 μm (**A**).

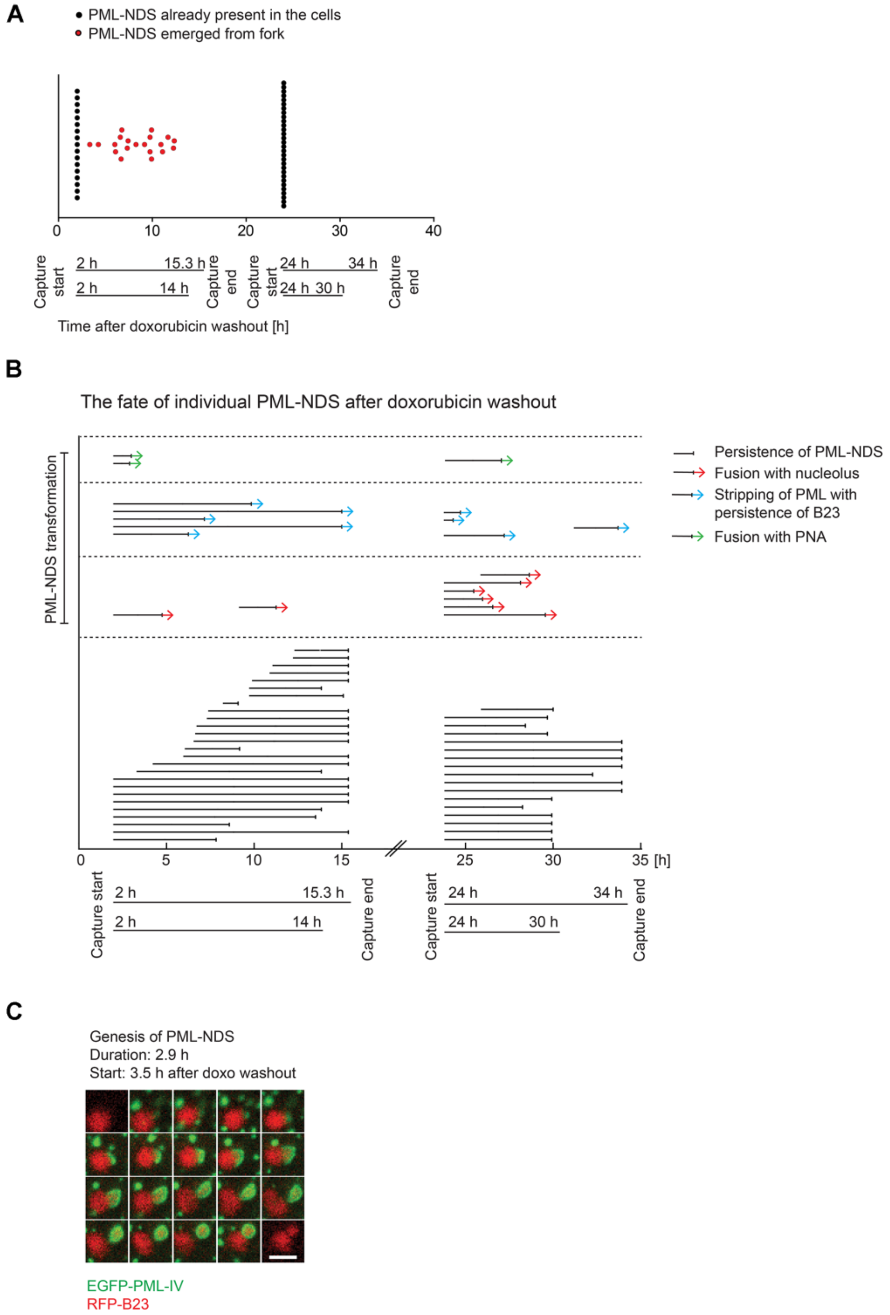

Supplementary Figure 7. PML-NDS originate preferentially from PML-nucleolar forks in early time-points after doxorubicin removal. (**A**) Quantitative analysis of the genesis of PML-NDS in RPE-1^{hTERT} stably expressing EGFP-PML IV and RFP-B23 after the treatment with 0.75 µM doxorubicin and the drug removal. The appearance of PML-NDS was followed by time-lapse microscopy in two sequential sessions from two independent experiments after the drug removal (experiment I: 2–15 and 24–34 hours; experiment II: 2–14 and 24–30 hours). PML-NDS that were already present in the beginning of the observation are marked by black dots; PML-NDS that emerged from forks during the observation time are marked by red dots and placed on the time scale according to the time when they emerged. (**B**) The genesis, stability and destiny of individual PML-NDS was followed in two sequential sessions from two independent experiments after the drug removal (experiment I: 2–15 and 24–34 hours; experiment II: 2–14 and 24–30 hours). The life-time of individual PML NDS is represented by solid lines with different endings marking the fates of PML-NDS: black line segment mark, end of capture session or cell escape from the view field; green arrow, fusion of PML-NDS with fork/cap; blue arrow, loss of PML but with persistence of B23; and red arrow, fusion of PML-NDS with nucleolus. (**C**) Time-lapse microscopy of the nucleolus illustrating the formation of PML-NDS. Note on the right side of the nucleolus, the whole transmutation cycle of PNAs was captured, i.e. the genesis of PML-cap on the border of the nucleolus, its transition into PML-fork, and finally into PML-NDS. EGFP-PML IV is shown in green, RFP-B23 in red. The initiation of capturing and the length of recorded time are given for each type of transition. Bar, 4 µm.

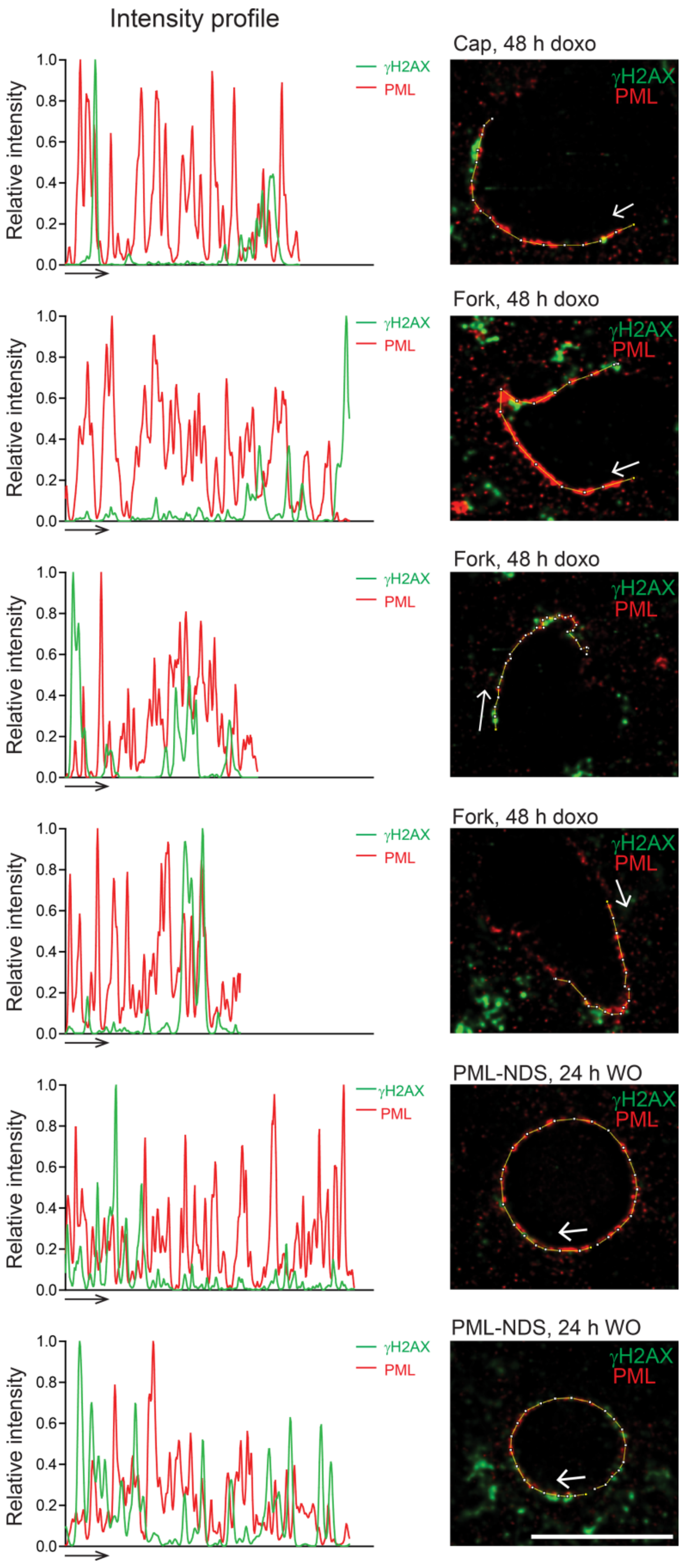

Supplementary Figure 8. PNAs associate with γH2AX. RPE-1^{hTERT} were treated with 0.75 µM doxorubicin for 2 days; after that doxorubicin was removed and cells were further cultivated. Super-resolution STED microscopy images of cells at different time-points are shown, and intensity profiles of γH2AX signal (green) and PML signal (red) at the border of each PNA are presented. The white and black arrows show the direction of intensity profile. Bar, 4 µm.

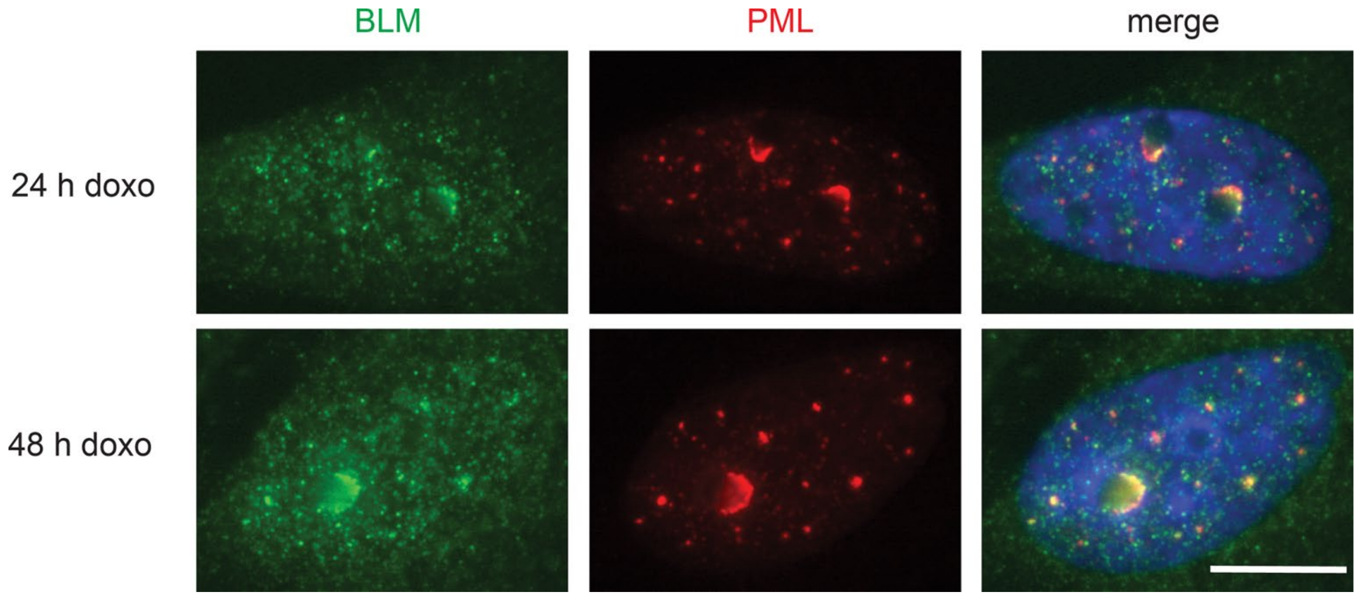

Supplementary Figure 9. BLM localizes to PNAs. RPE-1^{hTERT} were treated with 0.75 μM doxorubicin, harvested after 1 and 2 days and stained for PML (green) and BLM (red). The nuclei were counterstained with DAPI. The images were captured with 63×/1.4 objective. Bar, 10 μM.
