# Supplementary material for: Dynamic PML protein nucleolar associations with persistent DNA damage lesions in response to nucleolar stress and senescence-inducing stimuli
Source: Aging (Albany NY). 2019 Sep 7;11(17):7206–35. doi: 10.18632/aging.102248 (PMC6756913; doi:10.18632/aging.102248)
Supplement: Supplementary Table 1 [file aging-11-102248-s002.pdf]

SUPPLEMENTARY TABLE

Supplementary Table 1. Specification of antibodies

Antibodies	Specification	Cat. number	Company	Dilution
anti-BLM	rabbit, polyclonal		gift from dr. Norma F. Neff	1:100
anti-BrdU	mouse, monoclonal	B8434	Sigma-Aldrich/Merck (Darmstadt, Germany)	1:500
anti-Daxx	mouse, monoclonal	03	Exbio	1:100
anti-DHX-9	rabbit, polyclonal	NB110-40579	Novus Biologicals (Centennial, CO, USA)	1:400
anti-fibrillarin	rabbit, polyclonal	sc-25397	Santa Cruz Biotechnology (Dallas, TX, USA)	1:500
anti-PML	mouse, monoclonal	sc-966	Santa Cruz Biotechnology (Dallas, TX, USA)	1:200
anti-PML	rabbit, polyclonal	sc-5621	Santa Cruz Biotechnology (Dallas, TX, USA)	1:200
anti-phosphoserine 139 of histone H2AX	mouse, monoclonal	05-636	Millipore/Merck (Darmstadt, Germany)	1:500
anti-nucleophosmin/B23	mouse, monoclonal	32-5200	Invitrogen/Thermo Fisher Scientific (Waltham, MA, USA)	1:200
anti-nucleolin/C23	rabbit, polyclonal	ab70493	Abcam (Cambridge, UK)	1:200
anti-PAF49	rabbit, polyclonal	ab92428	Abcam (Cambridge, UK)	1:250
anti-Sp100	rabbit, polyclonal		gift from prof. Pavel Hozák	1:200
anti-SUMO1	rabbit, polyclonal	ab32058	Abcam (Cambridge, UK)	1:300
Alexa Fluor 488 goat anti-mouse	goat anti-mouse	A-11029	Invitrogene/Thermo Fisher Scientific (Waltham, MA, USA)	1:1000
Alexa Fluor 568 goat anti-mouse	goat anti-mouse	A-11031	Invitrogene/Thermo Fisher Scientific (Waltham, MA, USA)	1:1000
Alexa Fluor 488 goat anti-rabbit	goat anti-rabbit	A-11034	Invitrogene/Thermo Fisher Scientific (Waltham, MA, USA)	1:1000
Alexa Fluor 568 goat anti-rabbit	goat anti-rabbit	A-11036	Invitrogene/Thermo Fisher Scientific (Waltham, MA, USA)	1:1000
Alexa Fluor 555 goat anti-rabbit	goat anti-rabbit	A-21434	Invitrogene/Thermo Fisher Scientific (Waltham, MA, USA)	1:1000
